# Dissolving Microneedles Containing Lactoferrin Nanosuspension for Enhancement of Antimicrobial and Anti-Inflammatory Effects in the Treatment of Dry Eye Disease

**DOI:** 10.3390/pharmaceutics17050653

**Published:** 2025-05-16

**Authors:** Sammar Fathy Elhabal, Ahmed Mohsen Faheem, Sandra Hababeh, Jakline Nelson, Nahla A. Elzohairy, Suzan Awad AbdelGhany Morsy, Tassneim M. Ewedah, Ibrahim S. Mousa, Marwa A. Fouad, Ahmed Mohsen Elsaid Hamdan

**Affiliations:** 1 Department of Pharmaceutics and Industrial Pharmacy, Faculty of Pharmacy, Modern University for Technology and Information (MTI), Mokattam, Cairo 11571, Egypt; 2 Department of Medical Biochemistry and Molecular Biology, Faculty of Medicine, Mansoura University, Mansoura 35516, Egypt; amfaheem@fcms.edu.sa; 3 Department of Pharmaceutics, College of Pharmacy, King Saud University, Riyadh 11451, Saudi Arabia; sandra.hababeh@outlook.sa; 4 Department of Microbiology and Immunology, Faculty of Pharmacy, Nahda University, Beni-Suef (NUB), Beni-Suef 62511, Egypt; jaqueline.nielsen@nub.edu.eg; 5 Department of Microbiology and Immunology, Faculty of Pharmacy, Modern University for Technology and Information (MTI), Mokattam, Cairo 11571, Egypt; nahla.elzohairy@pharm.mti.edu.eg; 6 Air Force Specialized Hospital, Cairo 19448, Egypt; 7 Department of Clinical Pharmacology, Faculty of Medicine, Alexandria University, Dr. Fahmi Abdelmeguid St., Mowassah Campus, Alexandria 21561, Egypt; susan.ulghani@alexmed.edu.eg; 8 Pathological Sciences Department, MBBS Program, Fakeeh College for Medical Sciences, Jeddah 21461, Saudi Arabia; 9 Pharmaceutics and Pharmaceutical Technology Department, Faculty of Pharmacy, Egyptian Russian University, Cairo 11829, Egypt; tassneim-mohammed@eru.edu.eg; 10 Pharmaceutics Department, Faculty of Pharmacy, Sinai University, Al-Arish 45511, Egypt; ibrahim.salah@su.edu.eg; 11 Department of Pharmaceutics and Pharmaceutical Technology, Faculty of Pharmacy, Deraya University, Minia 61768, Egypt; marwa.fouad@deraya.edu.eg; 12 Department of Pharmacy Practice, Faculty of Pharmacy, University of Tabuk, Tabuk 71491, Saudi Arabia

**Keywords:** dry eye disease (DED), methicillin-resistant *Staphylococcus aureus* (*MRSA*), Schirmer tear test, Draize test, nanosuspension

## Abstract

**Background/Objectives:** Dry eye disease (DED), also known as “keratoconjunctivitis sicca”, is a common chronic ocular surface disease accompanied by inflammation and diminished tear production. Bovine Lactoferrin (BLF), a multi-functional iron-binding glycoprotein found in tears, decreased significantly in patients with DED, used for the treatment of dry eye, conjunctivitis, and ocular inflammation. BLF has limited therapeutic efficacy due to poor ocular bioavailability. **Methods:** This study developed and optimized a BLF-loaded nanosuspension (BLF-NS) using the Box–Behnken Design (BBD). Optimized BLF-NS was then incorporated with polyvinyl pyrrolidone (PVP) and hydroxypropyl methyl cellulose (HPMC) dissolving microneedles (MNs). The formulations were characterized by Scanning and transmission microscopy, DSC, FTIR, ex vivo studies in corneal tissue from sheep and tested for its antibacterial and antifungal efficacy against Methicillin-Resistant *Staphylococcus aureus* (*MRSA*), *Staphylococcus aureus*, and *Aspergillus niger*, respectively. Moreover, they were tested for their Benzalkonium chloride (BCL) dry eye in a rabbit model. **Results:** The optimized nanosuspension showed a vesicle size of (215 ± 0.45) nm, a Z.P (zeta potential) of (−28 ± 0.34) mV, and an Entrapment Efficiency (EE%) of (90 ± 0.66) %. The MNs were fabricated using a ratio of biodegradable polymers, PVP/HPMC. The resulting BLF-NS-MNs exhibited sharp pyramidal geometry with high mechanical strength, ensuring ocular insertion. In vitro release showed 95% lactoferrin release over 24 h, while ex vivo permeation achieved 93% trans-corneal delivery. In vivo, BLF-NS-MNs significantly reduced pro-inflammatory cytokines (TNF-α, IL-6, MMP-9, IL-1β, MCP-1) and upregulated antioxidant and anti-inflammatory genes (PPARA, SOD 1), restoring their levels to near-normal (*p* < 0.001). **Conclusions:** The nanosuspension combined with MNs has shown higher ocular tolerance against DED ensured by the Draize and Schirmer Tear Test.

## 1. Introduction

Dry eye disease (DED) is a persistent polygenic condition that impacts the ocular surface, marked by inadequate tear production from the tear glands [1]. The conditions are classified into two categories: aqueous-deficient and hyper-evaporative dry eye conditions. Furthermore, the “Tear Film and Ocular Surface Society Dry Eye Workshop II 2007” defined dry eye disease [2]. DE impacts half of the global population and incurs an annual cost ranging from USD 687 to USD 1267 per individual. Regular drug administration, ocular surgery, prolonged screen exposure, contact lens usage, and specific environmental factors have all played a role in the increased incidence of dry eye [3]. Dry eyes can cause corneal stress, inflammation, and instability of the tear film. Reduced mucosal protection on the ocular surface has increased the risk of corneal infection and ulceration [4,5]. The lacrimal gland, eyelids, meibomian gland, and tear film work together to keep the ocular surface properly lubricated. Meanwhile, reduced aqueous and mucosal layers on the ocular surface resulted in tear film dysfunction in DE [6]. This mechanism activated T-lymphocytes on the ocular surface, causing irritation and redness. Furthermore, DE caused severe ocular pain, redness, blurred vision, and itchiness in the eye, which reduced functional vision [7]. Cyclosporine A (Restasis), Lifitegrast (Xiidra), Tetracyclines (Doxycycline, Minocycline), Corticosteroids (Prednisolone, Fluorometholone), Artificial Tears (Lubricants), Autologous Serum Eye Drops, Secretagogues (Pilocarpine, Cevimeline), and Bovine Lactoferrin can treat DE [8]. Dry eye can be treated with warm compresses for the meibomian glands, nasolacrimal gland stimulation, contact lenses, surgery, intravitreal injections, and topical treatments [5,9].

Bovine Lactoferrin (BLF), an 80–90 kDa glycoprotein that binds to iron, is a mammalian, first-line defense, multifunctional protein from the transferrin family with the unique ability to bind ferric iron [10]. It is constitutively expressed in mucosal surfaces and secretory fluids, particularly milk, but also the digestive tract, bile, saliva, bronchial and nasal secretion, cervicovaginal mucus, seminal fluids, tears, and mucosal secretions. BLF regulates the immune system, fights infections, and heals tissue by binding iron [11]. Lactoferrin, found in milk, is antimicrobial and anti-inflammatory. In breast cancer, BLF modulates immune responses and regulates immunity to bacteria, fungi, and viruses. Even in high doses, it is safe. Its many uses have sparked interest in cancer, inflammatory disease, and infection treatments [12]. Prostate cancer patients express more transferrin receptors, suggesting that lactoferrin can target prostate tumor cells. Macrophages and neutrophils catch and kill pathogens with immunomodulatory lactoferrin. Extensive studies show that lactoferrin kills viruses, fungi, and bacteria, causing membrane damage [13]. Collagen, a necessary extracellular matrix component, is produced by active fibroblasts, which encourages tissue regeneration. This mechanism modulates inflammatory responses, including TNF-α, IL-1, and IL-6, as well as cytokine synthesis. It is antiviral and antibacterial since its iron affinity neutralizes viruses and stunts bacterial development [14]. Lactoferrin lowers inflammation, thereby slowing down wound healing; it also stimulates angiogenesis, which generates blood vessels to feed healing tissue oxygen and nutrients. For diabetics, high serum BLF levels could help to reset their immune systems [15]. Lactoferrin’s cancer treatment possibility has lately attracted attention in research. Along with changing immune cells to fight cancer, it causes death in cancer cells, thereby lowering tumor size and metastasis. Lactoferrin also inhibits tumor angiogenesis, which is the formation of blood vessels that supply tumors. Lactoferrin is effective at fighting infections and improving oral gum health [16]. Its antimicrobial properties make it effective for treating gastrointestinal infections and inflammatory bowel diseases. Moreover, when utilized for ocular applications, BLF demonstrates efficacy in alleviating dry eye symptoms, minimizing inflammation, and ensuring no ocular irritation, as it rapidly exits via the conjunctiva and nasolacrimal duct [17]. Lactoferrin is mostly apo- and holo-lactoferrin, both of which bind iron. These biological activities may affect ocular surface disease treatment. For dry eye disease, inflammation and oxidative stress are key factors. Apo-lactoferrin’s affinity for chelating free iron limits iron-catalyzed ROS generation via the Fenton reaction. Reduced oxidative stress and protection of ocular surface epithelial cells are achieved through this antioxidant action. Additionally, apo-lactoferrin reduces ocular inflammation by suppressing pro-inflammatory cytokines like IL-6 and TNF-α. Holo-lactoferrin (iron-saturated form) has lower iron-binding capacity but may be more effective against certain pathogens by interfering with microbial iron acquisition mechanisms. However, its lower iron-chelating potential may provide less antioxidant protection than apo. Lactoferrin’s ability to modulate immune responses, promote epithelial healing, and stabilize the tear film may vary with iron saturation, according to studies. Apo-lactoferrin boosts mucosal immunity and epithelial proliferation, which helps restore ocular surface homeostasis in DED. Lactoferrin topical application has been shown in mouse models to reduce irradiation-induced corneal epithelial damage and promote corneal wound healing after alkali burn injury [18]. Furthermore, previous studies have found a link between low tear lactoferrin levels and the development of both dry eye disease (DED) and chronic meibomitis. Common ocular surface system diseases show inflammation and oxidative stress markers, so their pathophysiology is somewhat similar [19]. The rationale for using lactoferrin in DED treatment is based on its ability to directly address the disease’s vicious cycle, particularly underlying inflammation and oxidative stress. Lactoferrin has oxygen-free radical and hydroxyl scavenging properties because of its iron-chelating ability, which prevents reactive oxygen species from causing inflammation and tissue damage [20]. Lactoferrin, on the other hand, reduces inflammatory responses to pathogens by inhibiting classical complement activation and downregulating inflammatory mediators such as TNF-α, IL-1, IL-6, and IL-8, ICAM-1, and CD14 [21]. Patients supplemented with oral lactoferrin showed improved dry eye symptoms and tear film stability in a study by Dogru et al. [22], and another study reported its efficacy in improving ocular surface parameters such as tear break-up time and the Schirmer test in patients affected by dry eye caused by cataract surgery [20]. Furthermore, locally applied lactoferrin was able to restore corneal epithelial integrity in a rabbit model of dry eye, pointing out the potential use of lactoferrin eye drops for treating DED.

Our formulation used Bovine Lactoferrin (≥98% purity), which was not enriched for apo- or holo-form but would predominantly be in the apo-form under physiological pH and processing conditions. Apo-lactoferrin’s mechanisms likely explain the study’s anti-inflammatory and antioxidant effects. Topical medications represent some of the most frequently utilized therapies for DE. According to the biopharmaceutical classification system (BCS), BLF is a Class II compound with limited solubility and high permeability [23]. Meanwhile, marketed topical formulations have several limitations, including increased intraocular pressure, shorter retention time, and greater nasolacrimal drainage [24]. Nanosuspensions (NSs) serve as a valuable solution for the challenges posed by insoluble or poorly soluble drugs in aqueous environments while also catering to the demands of personalized medicine [25]. Nonetheless, NS particles may lack sufficient properties to navigate the physiological barriers associated with administration routes or the challenging biological environment, thereby diminishing the effectiveness of NS. The functionalization of the NS surface is achievable and serves as an effective approach to enhance targeted delivery, leading to the development of advanced delivery systems with tailored characteristics [26,27]. The coating of the NS may take place via electrostatic interactions, dipole–dipole interactions, hydrogen bonds, or hydrophobic interactions. Therefore, it is essential to consider certain structural characteristics of the polymers and the nanosuspension during the development of these systems [28]. Poloxamer 188 (P188), TPGS, and PEG were chosen as stabilizers and plasticizers because of their demonstrated ability to improve nanosuspension stability, solubility, and particle aggregation during formulation. TPGS, a vitamin E derivative, adds surfactant and antioxidant properties, whereas PEG enhances hydrophilicity and steric stability.

A microneedle (MN) is a small device with many tiny needles of different shapes on a base [5]. Microneedle height typically ranges from 25 to 2000 µm. Microneedles are used in drug delivery systems because they avoid first-pass metabolism, improve drug bioavailability, improve skin delivery, are painless, increase patient compliance, and control drug release [29]. Microneedles pierce the skin to create multiple micro-holes for drug or large substance delivery. Four types of microneedles exist: solid, hollow, coated, and dissolving. Hyaluronic acid, chondroitin sulfate, polyvinyl pyrrolidone (PVP), and hydroxypropyl methyl cellulose (HPMC) are water-soluble matrix materials in dissolving microneedles, which contain therapeutic compounds [30]. If biological fluid touches dissolving microneedles on the skin, they dissolve and release loaded therapeutic compounds directly to the therapeutic site. PVP and HPMC are skin-insertable, biodegradable, and non-toxic polymers. Dissolving microneedles made of PVP and/or HPMC deliver insulin, lidocaine, and alpha-arbutin [31,32]. PVP and HPMC dissolving microneedles may deliver therapeutic or bioactive compounds for hair loss or alopecia. Previous research suggests nanosuspensions (NSs) can address drug delivery issues with hydrophobic drugs. Unlike lipidic or polymeric nanoparticles, NS drug molecules, as pure nanosized forms with minimal surfactants, result in high drug loadings for loading BLF into dissolving MN arrays; NSs were introduced in this work.

A previous study investigated the parameters in microneedle patch preparation that affect the appearance of the PVP/HPMC microneedle, as well as the effects of various shapes on the microneedle’s properties. Still, neither the skin penetration nor the features of microneedles, including active compounds, were investigated [32]. In the current study, we used 3D printing to fabricate and characterize dissolving microneedles made of polyvinylpyrrolidone K90 (PVP-K90) and hydroxypropyl methylcellulose E50. The selected formulation of dissolving microneedle that exhibited proper physicochemical properties was incorporated into the *Oryza sativa* L. extract complex to investigate its characteristics, transfollicular penetration, and safety [33].

This study aimed to develop and optimize a lactoferrin-loaded nanosuspension (BLF-NS) to improve the therapeutic efficacy of Bovine Lactoferrin (BLF) in the treatment of dry eye disease (DED). Using a solvent-anti-solvent precipitation technique, the work began with the formulation of BLF-NS, optimizing key parameters such as particle size, zeta potential, and drug content using the Box–Behnken design. The optimized BLF-NS was then integrated into dissolved microneedles (BLF-NS-MNs) made of PVP and HPMC. The new BLF-NS-MN formulation aims to improve ocular bioavailability, facilitate penetration through the corneal epithelium, and extend drug retention by addressing the issues of low bioavailability and short ocular retention time commonly associated with conventional formulations. The microneedle-based system showed improved anti-inflammatory, antibacterial, and antifungal effects, greatly improving the expression of important inflammation-related genes, including COX-2, PPARA, and SOD 1 and lowering pro-inflammatory cytokines, including TNF-α, IL-6, MMP-9, IL-1β, and MCP-1. These findings show that combining microneedle technology with nanosuspension provides sustained drug release, improved drug delivery, and efficient relief from ocular inflammation and oxidative stress, thus offering a promising new approach for treating DED.

## 2. Materials and Methods

### 2.1. Materials

Bovine Lactoferrin (BLF, ≥98% purity) was obtained from Sigma Aldrich Pvt. Ltd. (Darmstadt, Germany). Poloxamer 188 (pharmaceutical grade), D-α-tocopheryl polyethylene glycol succinate (TPGS, ≥99% purity), propylene glycol (PG, ≥99.5%), polyethylene glycol (PEG, average M.W. 400, ≥99%), and polyvinylpyrrolidone (PVP, K30 grade) were purchased from Acofarma (Madrid, Spain). Hydroxypropyl Methylcellulose (HPMC, Methocel E15 LV), phosphotungstic acid (≥99%), and potassium bromide (KBr, FTIR grade) were procured from BASF (Ludwigshafen am Rhein, Germany). Ethanol (≥99.9%), distilled water, and acetonitrile (HPLC grade) were obtained from Merck Life Sciences. All solvents were of analytical or chromatography grade and used without further purification unless stated otherwise.

### 2.2. High-Performance Liquid Chromatography Method of Lactoferrin (BLF)

High-Performance Liquid Chromatography (HPLC) analysis was performed using a C18 column (reverse-phase) measuring 250 mm × 4.6 mm and a particle size of 5 µm (Phenomenex, Torrance, CA, USA). The mobile phase is made up of sodium acetate buffer and acetonitrile in a 40:60 ratio, mixed with an acetate buffer with a pH of 7.4 and flowing at 1.0 mL/min [34,35]. To ensure sink conditions and prevent external auto-concentration during sampling, 200 μL samples were collected and replaced with pre-warmed PBS pH 7.4 after each sampling time. UV detection revealed a correlation coefficient of 0.9998 (Waters 2487, Waters Corporation, located in Milford, MA, USA). At the maximum absorption wavelength for BLF amounts at 220 nm, the cumulative amounts of BLF versus time were calculated.

### 2.3. Preparation of Lactoferrin Nanosuspensions

**Figure 1:**
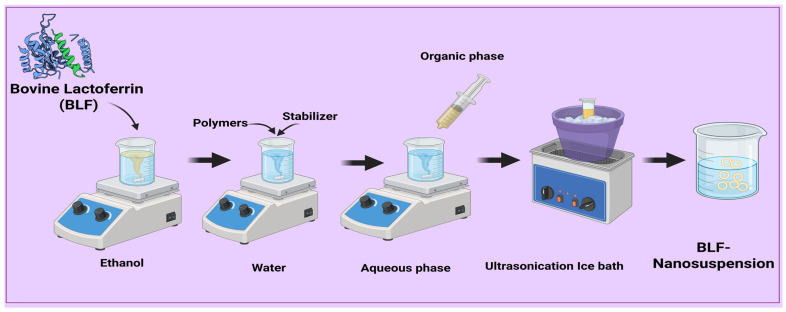
Diagram illustrating the methodology for preparing lactoferrin nanosuspensions.

A solvent-anti-solvent precipitation method produced lactoferrin nanosuspension. BLF (10 mg) was dissolved in a suitable volume of organic solvent (ethanol) under a magnetic stirrer (SCHOTT, Mainz, Germany). Dissolving polymers and stabilizers in distilled water with a magnetic stirrer produced the anti-solvent phase. The organic solvent was then injected into the aqueous phase with a syringe and stirred continuously [36,37]. Following the precipitation of BLF nanoparticles, stirring continued for an additional 0.5, 1, or 2 h to ensure that the organic solvent was completely evaporated. To further lower the BLF particle size, the nanosuspension was then run through 10 min of ice-water ultrasonic (CPX 130, Cole-Parmer, Vernon Hills, IL, USA). To stabilize the nanosuspension, 3% mannitol was added to BLF-NS before pre-freezing at −80 °C and freeze-drying for 48 h, as shown in Figure 1. Based on their proven roles in stabilizing nanoparticle suspensions by lowering interfacial tension, preventing aggregation, and improving drug solubility, P188, TPGS, and PEG were selected as formulation variables. With their amphiphilic form, TPGS also aids in better drug encapsulation and bioavailability.

### 2.4. Optimization of Formulation

The 3^4^-factorial design and version 13 of Design Expert^®^ software (Stat-Ease, Minneapolis, MN, USA) were used to optimize formulation with BLF-NS, as shown in Table 1. The investigation focused on four variables. The factors are (A) Polymer Conc (*w*/*v*%), (B) Stirrer time (h), (C) Polymer Type, and (D) Plasticizer Type. Table 1 lists the Poly dispersibility index (Y2), zeta potential (MV) (Y3), drug content (%) (Y4), and particle size (nm) (Y1) as responses (dependent variables). The following optimization rules were used to make the Design Expert^®^ software (version 13). Particle size (P.S) and polydispersity index (P.D.I) were set to the lowest values possible to achieve the best nanosuspension stability and drug loading. Zeta potential (Z.P, absolute value) and Entrapment Efficiency (EE%) were set to the highest values possible. A statistical study was conducted using analysis of variance (ANOVA) at a 95% level of significance (*p* < 0.05) to estimate the data received and the impact of the variable on BLF-NS preparation.

### 2.5. Investigation of Lactoferrin-Loaded Nanosuspension

#### 2.5.1. Measurement of Particle Size, Polydispersity Index, and Zeta Potential

By using the dynamic light scattering (DLS) technique, the mean particle size, polydispersity index, and zeta potential were determined using a particle size analyzer (Horiba Scientific SZ-100, a division of HORIBA, Ltd., headquartered in Kyoto, Japan). Freshly prepared NS was diluted 100 times with distilled water and analyzed [38,39].

#### 2.5.2. Drug Content Analysis of Lactoferrin Nanosuspension

All freshly prepared nanosuspensions were centrifuged at 10,000 rpm at 25 °C for 10 min using a centrifuge (Model Z 300 K, Hermle Labortechnik Gmbh, Wehingen, Germany). To determine the free drug, the supernatant was analyzed using reverse-phase HPLC, as described in Section 2.2. Drug content was calculated using the following equation [40,41]:

(1) $$Drug\;content=(\frac{W_{initial}\;drug-W_{free}\;drug}{W_{initial}\;drug})\times 100$$

where W_initial_ drug = weight of the initial drug added and W_free_ drug = weight of the free drug in the supernatant.

### 2.6. Characterization of Lactoferrin Nanosuspension

#### 2.6.1. Transmission Electron Microscopy (TEM)

The samples were positioned on a copper mesh coated with a carbon film and permitted to dry at ambient temperature, followed by negative staining using phosphotungstic acid at a concentration of 2.0% (*w*/*v*). The transmission electron microscope (TEM) (JEM 1200EX, JEOL Co., Ltd., Tokyo, Japan) was employed to examine the surface morphology of Bre-NS and Bre-NS freeze-dried powder samples [42].

#### 2.6.2. Fourier Transform Infrared Spectroscopy (FT-IR)

The FT-IR (Nicolet IS50 Fourier transform infrared spectrometer, Waltham, MA, USA) was employed to simultaneously analyze BLF, the NS blank devoid of BLF, and the freeze-dried powder of BLF-NS. The samples were amalgamated with KBr and subsequently compressed at a pressure of 10 MPa into disks with a diameter of 10 mm. Total infrared spectra were acquired using Fourier transform infrared spectroscopy (FT-IR) at a resolution of 2 cm^−1^ within the wave number range of 400–4000 cm^−1^ [43,44].

#### 2.6.3. Differential Scanning Calorimetry (DSC)

Using a DSC (Q100, TA Co., Ltd., New Castle, DE, USA), the thermal characteristics of BLF, NS blank without BLF, and BLF-NS freeze-dried powder were ascertained. The samples were arranged on a sealed aluminum tray covered in perforations. With a heating rate of 10 °C per minute, the measured temperature range ran from 10 to 300 °C. Measurements were conducted under 50 mL/min nitrogen flow. Measurements were taken with a nitrogen flow of 50 mL/min. Measurements were made under a nitrogen flow of 50 mL/min [45,46].

#### 2.6.4. Drug Release Studies

##### In Vitro Drug Release Q24 of Lactoferrin from BLF-NS

In vitro dissolution and release studies of BLF suspension and BLF-NS were performed using Pharma Test dissolution tester type II (Paddle Apparatus, SP6-400; Hamburg, Germany). A total of 500 mL of phosphate buffer pH 6.8 with 1% Tween 80 was used as a release medium and maintained at 37 ± 0.5 °C and 100 rpm. In Float-A-Lyzer cellulose ester dialysis tubes (1 mL, molecular weight cut-off of 10 kDa, Spectrum Laboratories, Los Angeles, CA, USA), at predetermined intervals, aliquots of the release medium containing 1 milliliter were extracted and replaced with fresh PBS of the same volume. As previously discussed in Section 2.2, the amount of BLF released from BLF suspension and BLF-NS release was measured using HPLC for up to 24 h [47,48]. The experiment was repeated three times to ensure accuracy.

##### Ex Vivo Permeation Study

Ex vivo penetration in corneal tissue from sheep was acquired from a nearby Cairo, Egypt, slaughterhouse. Ex vivo evaluation of ocular penetration of various BLF formulations was performed using a modified Franz diffusion cell device. The surface of the sheep’s cornea was positioned in the diffusion cell, and an ocular BLF solution equivalent to the drug, BLF-NS, along with BLF-NS-MNs, was sprayed onto it. A Teflon-coated magnetic stir bar placed at the bottom of the receptor cell facilitated uniformity in the receptor volume while water kept at 37 °C flowed through the water jacket encasing the receptor cell. Phosphate buffer at a pH of 7.4 made up the diffusion medium. The concentration of BLF was quantified after a sample of receptor media was gathered at designated intervals (1, 2, 4, 6, 8, 24, 48, 72, and 80 h). The cornea was removed from the cell after eighty hours and homogenized for five minutes using five milliliters of DMSO [44,49].

#### 2.6.5. Stability Study

Nanosuspensions were stored at 4 ± 2 °C for six months to assess short-term stability. Vesicle dimensions, polydispersity index (P.D.I), and zeta potential were determined at predetermined intervals (0 and 3 months) using the methods described in Section 2.2. Measurements were taken three times to ensure reliability, and results were reported as mean ± standard deviation (SD) [50,51].

### 2.7. Fabrication of PVP/HPMC Microneedles Loading Freeze-Dried BLF-NS

Dissolving PVP K90 and HPMC E50 in a 70:30 solution of anhydrous ethanol and deionized water produced formulations 20:5, 15:15, and 5:25. Table 2 lists the formulations’ respective component quantities. After heating the solutions to 60 degrees Celsius, they swirled until they were homogeneous. The polymeric solutions were moved to centrifuge tubes; next, the design and production of polydimethylsilane (PDMS) (5 × 5 arrays, tip parameter H300 B100) was undertaken [5,30]. The solutions were centrifuged for 1 h at 8000 rpm at 25 °C using a centrifuge machine (MPW-352R, Warsaw, Poland) to ensure that the solution filled the mold cavity. The microneedle molds were then removed from the centrifuge tubes and heated at 45 degrees Celsius until the microneedles had dried at room temperature for 24 h. Finally, the dried microneedles were carefully extracted from the molds [52].

### 2.8. Microneedle Morphological Characterizations

#### 2.8.1. Physical Characterization of MN

The length, tip diameter, and base diameter of arrays of BLF-NS were measured under Raman Microscope (SENTERRA, BRUKER, Billerica, MA, USA).

#### 2.8.2. Mechanical Strength/Breaking Strength

Weights (250 gm, 500 gm, 1000 gm) were applied on the tips of the BLF-NS-loaded MN patch for five minutes, after which the length of the MN arrays was measured using the Raman Microscope (SENTERRA, BRUKER), indicating the mechanical strength of the microneedles [5,53].

#### 2.8.3. Drug Content Studies

At a stirring speed of 300 rpm, distilled water with 2.5% Tween 80 was employed to dissolve an array of BLF-NS-loaded magnetic agitators for 12 h. The resulting solution was diluted with methanol and sonicated for five minutes to ensure the complete dissolution of BLF from the created BLF-NS-loaded MNs. The drug content of the produced MNs was determined by HPLC analysis [5,54].

#### 2.8.4. Physicochemical Characterization for the Optimized MN

##### Scanning Electron Microscopy (SEM)

A scanning electron microscope (JEOL JSM-IT200, Pleasanton, CA, USA) was used to analyze the shape and morphology of the MN patch loaded with BLF-NS. To measure the needle morphology and dimensions, the MN patch was coated with gold and examined using the PhenomTM SEM system at 30.00 kV and 400× magnification [55].

##### Fourier Transform Infrared Spectroscopy (FTIR)

Fourier transform infrared spectroscopy spectra for Pure BLF, BLF-NS-MNs, and MNs Blank were obtained using the same method mentioned before.

##### Differential Scanning Calorimetry (DSC)

Differential Scanning Calorimetry thermograms for pure BLF, BLF-NS-MNs, and their MNs Blank were obtained using the same method mentioned before.

##### Drug Release Studies for Microneedles Formulation

In vitro drug release Q24 of Lactoferrin from BLF-NS-MNs:

To quantitatively estimate the in vitro drug release from the MN patch, we conducted an in vitro drug release using the same method mentioned before.

Ex vivo permeation study:

We tested the ex vivo penetrability of BLF, BLF-NS-MNs solutions for corneal tissue using the same method mentioned before.

### 2.9. Antimicrobial Study

#### 2.9.1. Diffusion Agar Method

The antimicrobial activity of the BLF and BLF-NS solutions was preliminarily qualitatively evaluated using standardized microbial strains Methicillin-Resistant *Staphylococcus aureus* (*MRSA*) ATCC 4330, *Staphylococcus aureus* ATCC 25923, and *Aspergillus niger* (RCMB 002005). The suspension of these strains with a calibrated concentration of 0.5 McFarland (1.5 × 10^8^ CFU/mL) was produced by incubating at 37 °C for 18 to 24 h. Afterward, solutions were integrated into sterile agar plates. Separate samples were attentively drawn out from ten microliters of various diluted formulations [43,56]. The agar plates were incubated at 37 °C for 24 h, following the established droplet adsorption protocol. The presence of an inhibitory zone around the application site suggested antibacterial efficacy. Each experiment was repeated three times in order to measure and document the diameter of the inhibitory zones. All results were analyzed in order to determine its clarity [42,57].

#### 2.9.2. Quantitative Determination of the Minimum Inhibitory Concentration (MIC)

The MIC was determined using the micro-dilution technique. In this experiment, the standardized strains of Methicillin-Resistant *Staphylococcus aureus*, *Staphylococcus aureus*, and *Aspergillus niger* were grown on Mueller–Hinton Agar (MHA) at 37 °C until they reached the exponential growth phase [42,58]. In order to serially dilute our formulations, 100 µL of the Regional Center for Mycology and Biotechnology (RCMB) was added to each well of the flat-bottomed 96-well microplates, except for the first row, which had 200 µL of the total volume of the solutions of the formulations. Results were expressed as mean ± SD.

### 2.10. In Vivo Study

#### 2.10.1. Animals

Thirty-six male albino rabbits with an average weight (2–2.5 kg) were kept in cages in controlled room temperature (22–25 °C) and controlled relative humidity (55%). Rabbits had 7 days left before starting any experimental trial, they were supplied with their standard meals and water. The rabbits were kept in a 12 h light/dark cycle and unfettered access to food and water. The Ethics Committee authorized the experiments, which were carried out under the Research Ethics Committee’s guidelines (NUB-025-042).

#### 2.10.2. Dry Eye Models

**Figure 2:**
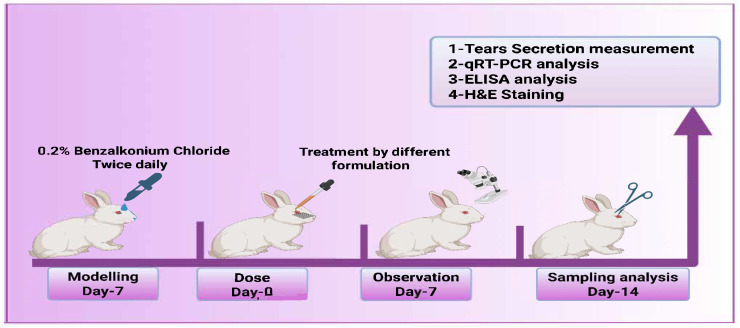
Schematic representation of an in vivo experimental study.

There were a total of thirty rabbits, divided into five groups (*n* = 6) that were used in a dry eye model. Group I left without induction of dry eye, while the other four groups induced dry eye by application of benzalkonium chloride (BCL) (25 μL, 0.2% *w*/*v*) injected twice daily (every 12 h) for seven days to develop dry eye syndrome. The rabbits then received treatment for one week according to the following classification: Group I: normal group (negative control), Group II: diseased group (positive control), Group III: dry eye treated with BLF solution, Group IV: dry eye treated with BLF-NS, and Group V: dry eye treated with BLF-NS-MNs [59] as shown in Figure 2.

#### 2.10.3. Eye Irritancy Test (Draize Test)

In order to evaluate the ocular irritancy of the following 0.9% NaCl (as a control solution), 0.05% benzalkonium chloride (BCL), BLF, and BLF-NS, 600 µg BLF-equivalent formulations were administered into the left eye (dropping into the conjunctival sac), while the right eye of the same animal rabbit administered 0.9% NaCl as a control. Prior to administration, all animals were anesthetized with 0.1 mg/kg butorphanol intramuscular injection. Ocular irritation parameters—including redness, tear secretion, and blink reflex—were assessed at predetermined time intervals following treatment. The ocular irritation score was recorded on a standardized Draize scale ranging from 0 (no irritation) to 4 (maximum irritation and redness) [60,61]. The scoring was performed by an independent observer who was blinded to the treatment groups to ensure objective and unbiased evaluation. Each eye was scored in duplicate by two blind evaluators, and discrepancies were resolved by consensus as shown in Figure 2.

#### 2.10.4. Schirmer Tear Test (Measurement of Tear Secretion)

Over five days, tear output was tracked with Schirmer Tear Test strips and Whatman No. 41 filter paper strips (Tianjin Jingming New Technological Development Co., Ltd., Tianjin, China). A total of twelve rabbits were used, divided into four groups. Using topical application of 0.005% benzalkonium chloride (BCL) for three months, three groups were induced with dry eye disease (DED), one group acting as the healthy control without any treatment. Following DED induction, treatment was given topically for five days straight using three different formulations: BLF, BLF-NS, and BLF-NS-MNs. Each group received its assigned treatment at consistent intervals. After treatment, rabbits were anesthetized with intramuscular butorphanol (0.1 mg/kg). Schirmer strips were gently inserted into the palpebral conjunctival sac of each rabbit eye for one minute to assess tear production. The wetted length of each strip was measured and compared between groups. The measurements were taken in triplicate at 30 min intervals [62,63].

#### 2.10.5. mRNA Expression in Cornea and Conjunctiva Detected by qRT-PCR

Conjunctiva tissues were used in order to extract the mRNA samples after their homogenization using 400 μL of lysate RZ. The lysates were incubated at room temperature for 5 min in order to fully dissociate the nucleic acid–protein complexes. Afterward, they were centrifuged at high speed (12,000 rpm) for 5 min at 4 °C. Afterward, the supernatant was transferred to a fresh tube that did not contain RNase [64]. The genetic expression levels of Peroxisome Proliferator-Activated Receptor Alpha PPAR-α (PPARA), Cyclooxygenase-2 (COX-2), and Superoxide Dismutase 1 (SOD 1) were quantified using the Quantitate SYBR Green PCR Kit (Cat. No. 204141, Qiagen, Hilden, Germany) in combination with specific primers, as shown in Table 3. The condition of the cycling process includes: the reverse transcription step, which took place at 50 °C for 30 min; the DNA denatured step at 94 °C for 15 min; and finally, the amplification step for 40 cycles, each cycle consisting of denaturation at 94 °C for 15 s, annealing at 60 °C for 30 s, and extension at 72 °C for 30 s [65,66]. A last extension step at 72 °C for 5 min concluded the operative process. Analysis was carried out by measuring the concentration of mRNA.

#### 2.10.6. Inflammatory Cytokines in Conjunctival Tissue Detected by ELISA

An enzyme-linked immunosorbent assay (ELISA) was performed in order to quantitatively assess the following: tumor necrosis factor-α (TNF-α), matrix metallopeptidase-9 (MMP-9), interleukin-1 beta (IL-1β), interleukin-6 (IL-6), and monocyte chemoattractant protein-1 (MCP-1) in the tissues of the rabbit eyes because the conjunctival tissues of the dry eye is frequently linked to elevated levels of inflammatory cytokines [70,71]. We aimed to quantitatively estimate the anti-inflammatory effect of BLF-NS and BLF-NS-MNs delivery systems through the reduction in the levels of the inflammatory cytokines during the treatment of dry eye syndrome.

### 2.11. Histopathological Examination

Conjunctiva and ciliary body samples were preserved in 10% neutral buffer formalin, then trimmed, washed with water, dehydrated using increasing concentrations of ethyl alcohol, cleared with xylene, and embedded in paraffin. Thin sections (4–6 µm) were then prepared and stained with hematoxylin and eosin (H&E) [72].

### 2.12. Statistical Analysis

To ensure validity, each experimental group included at least three replicates. Data were presented as mean ± SD. Statistical significance was assessed using one-way ANOVA, with a significance threshold set at *p* < 0.05, *** *p* < 0.001; ** *p* < 0.01; * *p* < 0.05.

## 3. Results and Discussion

### 3.1. Formulation Optimization of Lactoferrin Nanosuspension: Entrapment Efficiency (EE%), Particle Size (P.S), Polydispersity Index (P.D.I), and Zeta Potential (Z.P)

To achieve the best formulation parameters, lactoferrin nanosuspension (LF-NS) was developed and optimized systematically using a Box–Behnken Design

(BBD). In this study, the anti-solvent precipitation method was used to prepare formulations, with version 13 of Design Expert^®^ software generating 24 experimental runs. The four critical factors considered were polymer concentration (% *w*/*v*), stirring time (hours), polymer type (P188, TPGS, or Soy lecithin), and plasticizer type (PG, PEG, or Glycerol). These factors were compared to four key response variables: particle size (P.S), polydispersity index (P.D.I), zeta potential (Z.P), and drug content, as shown in Table 4. The optimization results revealed significant variations between experimental runs, emphasizing the importance of each factor in determining the LF-NS’s final properties.

The particle sizes varied greatly, ranging from 215 nm to 650 nm. The smallest particles were obtained in Run 18 (4% P188, PEG plasticizer, and 2 h stirring time), with a particle size of 215 ± 0.45 nm, which is a critical parameter for effective corneal penetration in ocular drug delivery.

In Run 18, the lowest recorded P.D.I value was 0.21 ± 0.06, with values ranging from 0.20 to 0.87. This indicates a high level of homogeneity, which is critical for ensuring consistent medication release and lowering the risk of aggregation during formulation storage. Soy lecithin and PEG plasticizer collaborated to produce a highly effective P.D.I.

**Figure 3:**
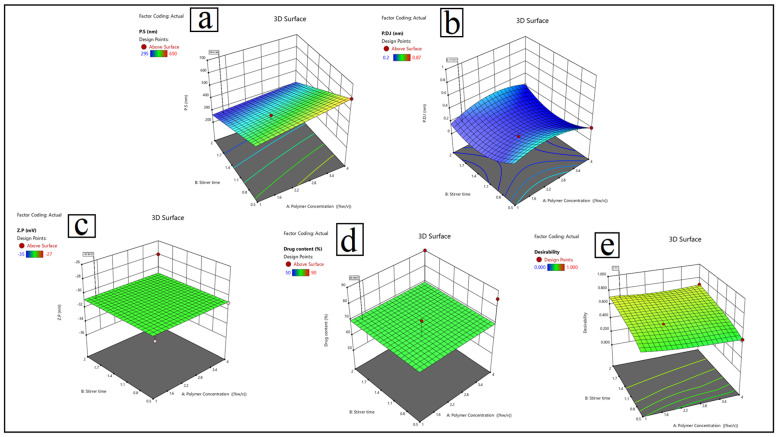
Response 3D plots for the effect of polymer concentration (A: *w*/*v*%), stirring time (B: hours), polymer type (C: P188, TPGS, or soy lecithin), and plasticizer type (D: PEG, PG, or glycerol), on (**a**) Particle size, (**b**) Polydispersity index, (**c**) Polydispersity index, (**d**) zeta potential, and (**e**) drug content (%).

Zeta potential values at or below −30 mV, specifically ranging from −27 mV to −35 mV, indicate optimal electrostatic stability, thereby preventing particle aggregation due to charge repulsion as shown in Figure 3.

Run 18, with 4% P188, PEG plasticizer, and a 2 h stirring time, had the highest drug content at 90%. This formulation had the highest drug loading capacity of all tested formulations, maximizing therapeutic potential. The quadratic model used for optimization had high predictive accuracy, with R^2^ values of 0.95 for particle size, 0.88 for P.D.I, 0.91 for Z.P, and 0.93 for drug content. The ANOVA results confirmed the model’s fit, with highly significant *p*-values (<0.0001) and no significant lack-of-fit (*p* > 0.05) for all responses.

- Particle size (Y1): Y1 = 420 + 25A − 30B − 15C + 10D − 12AB + 8AC − 5BC + 18A^2^.
- Polydispersity index (Y2) is calculated as: 0.25 + 0.08A − 0.05B − 0.12C + 0.03D + 0.02AB − 0.04AC + 0.01BC − 0.06A^2^ + 0.04B^2^.
- Zeta potential (Y3) is calculated as: Y3 = −28.5 + 1.2A − 2.8B + 3.5C − 1.8D + 0.9AB − 1.2AC + 0.5BC + 0.7A^2^ − 1.1C^2^
- Drug content (Y4) = 75 + 5A + 3B − 8C + 12D − 4AB + 7AD.

The validation of the optimal formulation exposed a great degree of agreement between experimental and expected values. The predicted particle size was 215 nm, which matched the actual value of 245 ± 5.2 nm. Moreover, the experimental results quite strongly correlated with the forecasts for P.D.I, zeta potential, and drug content. This validates the BBD model’s ability to scale up LF-NS production. PEG plasticizers mixed with P188 or soy lecithin polymers consistently resulted in formulations with high drug loading and stability. Higher polymer concentrations (4% *w*/*v*) reduced particle size, but careful optimization was required to keep P.D.I values within acceptable limits. The Box–Behnken Design (BBD) model was extremely effective at predicting formulation outcomes and is critical for increasing LF-NS production for dry eye treatment [73,74]. The optimized LF-NS formulation achieves an ideal balance of stability, particle size, and drug loading capacity, making it an excellent choice for ocular drug delivery applications. The BBD model’s high predictive capability and reliability make it ideal for translational pharmaceutical development.

### 3.2. Physicochemical Characterization of Lactoferrin Nanosuspension

#### 3.2.1. Transmission Electron Microscopy (TEM)

**Figure 4:**
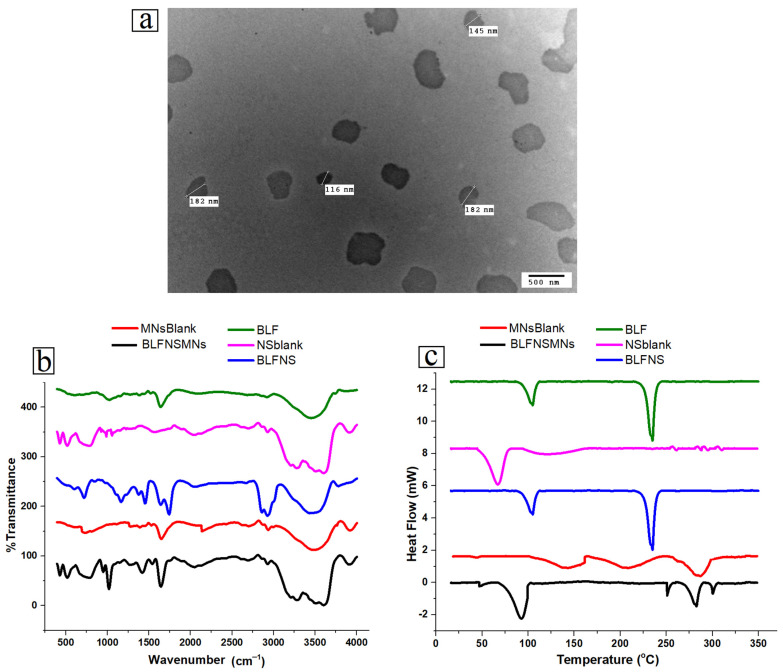
(**a**) Transmission electron microscopy, (**b**) Fourier transform infrared (FTIR) spectroscopy, and (**c**) Differential Scanning Calorimetry for BLF formulations. Note: BLF: Lactoferrin, NS-blank: nanosuspension without BLF, BLF-NS: Lactoferrin nanosuspension, MNs-blank: Polyvinylpyrrolidone, with Hydroxypropyl Methylcellulose, and BLF-NS-MNs: Lactoferrin nanosuspension loaded microneedles (MNs3).

The TEM results shown in Figure 4a indicate that the particle size of BLF-NS was 116–182 nm, and the TEM results had a relatively rounded, spherical-like structure with a uniform distribution, clear edges, and no adhesive aggregation. The reduction in particle size in TEM due to the hydration shell and the dispersed state of the particles when comparing particle sizes obtained from DLS and dry imaging methods like TEM [42,75].

#### 3.2.2. Fourier Transform Infrared Spectroscopy (FT-IR)

Figure 4b shows the FTIR to evaluate the structural integrity and chemical interactions of different lactoferrin formulations, namely BLF, NS blank, and BLF-NS. This research examines the functional groups and molecular interactions present in these formulations, which are essential for the stability and efficacy of the drug delivery system. The FTIR spectrum of pure BLF showed clear peaks that corresponded to the functional groups found in proteins. Proteins typically exhibit amide I bands at 1650 cm^−1^ and amide II bands at 1540 cm^−1^, representing C=O stretching and N-H bending vibrations, respectively. The presence of a broad band at 3300 cm^−1^ signifies N-H stretching, thus affirming the existence of the protein’s secondary structure [76,77]. The FTIR spectrum of BLF-NS exhibited peaks akin to those of BLF, accompanied by slight shifts noted in the amide I and amide II bands. The observed shifts suggest that interactions between the solvent and formulation may induce subtle conformational alterations in lactoferrin during the preparation of nanosuspensions. The distinct protein peaks indicate that lactoferrin maintains its secondary structure following nanosuspension processing, even in light of alterations in the formulation. The FTIR peaks of lactoferrin nanosuspension (BLF-NS) were strikingly similar to those of BLF after processing, indicating that the original structure was maintained. Nuanced differences or extensions of the amide I and amide II bands may indicate minor conformational changes in secondary structures during the preparation process. A strong band at ~1100 cm^−1^ (C-O-C stretching of P188/PEG), a peak at 1730 cm^−1^ (C=O stretching), and a broad band around 3400 cm^−1^ (O-H stretching) were revealed by the FT-IR spectrum of the NS-blank (nanosuspension without BLF). The BLF-NS spectrum included protein-specific amide I (~1650 cm^−1^) and amide II (~1540 cm^−1^) bands, indicating effective BLF incorporation. When comparing BLF-NS to pure BLF, the amide peaks are slightly moved and widened. This shows that there are mild BLF-polymer interactions instead of just overlapping polymer peaks. Figure 4b displays the FT-IR spectrum of pure BLF, which shows amide I and II bands at 1650 and 1540 cm^−1^, respectively. The NS-blank spectrum shows peaks at 1730 cm^−1^ (C=O stretching), 1100 cm^−1^ (C-O-C stretching), and a broad peak at 3400 cm^−1^ (O-H stretching), which indicate excipients. The BLF-NS spectrum (Figure 4b) demonstrates that polymer-related peaks overlap with BLF peaks. However, a slight shift in the amide I band to 1645 cm^−1^ and amide II to 1535 cm^−1^ indicates mild interactions between BLF and the polymer matrix. The intensity of the amide peaks was slightly lower in BLF-NS than in pure BLF, indicating hydrogen bonding or electrostatic interactions. The changes observed can be linked to the creation of nanosuspensions resulting from the interaction between lactoferrin and the solvent or the stabilizing agents in the formulation. Despite these slight variations, the structural integrity of lactoferrin remains the key factor affecting its effectiveness. FTIR studies conducted with nanosuspension formulations indicate that lactoferrin’s functional groups remain intact. The effectiveness of ocular drug delivery hinges on formulation retention, which is crucial for ensuring bioavailability, facilitating drug release, and enhancing efficacy.

#### 3.2.3. Differential Scanning Calorimetry (DSC)

Figure 4c shows Differential Scanning Calorimetry(DSC) to assess the thermal properties and stability of various lactoferrin formulations, including pure BLF, NS-blank, and BLF-NS. Pure BLF’s spectrum showed an endothermic peak in the 80–85 °C range, indicating denaturation or melting. High temperatures often lead to proteins losing their structural integrity, which disrupts their secondary structure. The absence of additional peaks indicates that the protein remains stable and intact, confirming the thermal stability of pure lactoferrin before denaturation. The NS-blank curve exhibited a broad endothermic peak in the range of 100 to 150 °C, indicating a thermal transition affected by excipients like P188 and PEG. The peak represents the glass transition or dehydration of the polymer matrix, indicating that the heating process affects the excipients. The lack of protein denaturation peaks in the NS-blank curve indicates the absence of lactoferrin, confirming that the formulation is polymeric [78]. The lactoferrin nanosuspension had similar thermal behavior to BLF, but there were some significant differences. The denaturation peak of lactoferrin shifted and broadened, indicating that its incorporation into the nanosuspension affected its thermal stability. This could be explained by protease–solvent interactions and stabilizing agents like P188 and PEG. Despite minor conformational changes caused by interactions with the excipient polymers, the DSC analysis of the lactoferrin formulations reveals that the lactoferrin protein maintains its thermal stability in nanosuspension. These changes are common when proteins are introduced into drug delivery systems, implying that lactoferrin is stable and functional in nanosuspension formulations.

#### 3.2.4. Drug Release Studies

##### In Vitro Drug Release Q24 of Lactoferrin from BLF-NS

**Figure 5:**
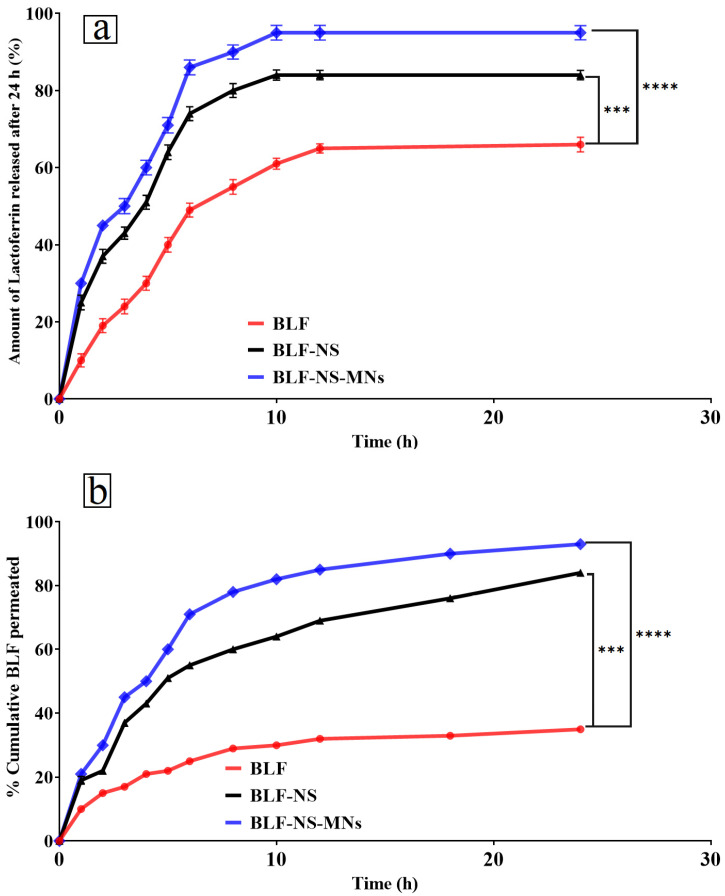
(**a**) In vitro drug release of lactoferrin over 24 h from pure BLF solution, BLF nanosuspension (BLF-NS), and BLF-loaded microneedles (BLF-NS-MNs), (**b**) Ex vivo permeation of lactoferrin across excised sheep cornea over 24 h from pure BLF solution, BLF nanosuspension (BLF-NS), and BLF-loaded microneedles (BLF-NS-MNs3). Note: Data represent mean ± SD (*n* = 3). Statistical significance indicated by *** *p* < 0.001, **** *p* < 0.0001 compared to pure BLF. The BLF-NS-MNs3 demonstrated significantly higher cumulative release and permeation versus pure BLF and BLF-Ns3.

Figure 5a shows the in vitro drug release profile of pure BLF, NS-blank, and BLF-NS. BLF release from these formulations was measured over 24 h to reveal drug release kinetics and formulation components’ effects on drug delivery. The BLF nanosuspension exhibited a sustained lactoferrin release profile, with only 36% released after 24 h. After a few hours of rapid release, the release rate slows. Pure lactoferrin lacks a controlled delivery matrix, so its sustained release is lower than nanosuspension or microneedle formulations. In targeted delivery for dry eye treatment, a sustained release may be better than a slow release. Blank nanosuspension under 2% drug release was observed throughout the 24 h NS-blank. This shows that lactoferrin-free nanosuspension does not release drugs [44,79]. It suggests that BLF-NS release is caused by lactoferrin and not by the nanosuspension itself. The BLF-NS formulation released the drug faster than pure lactoferrin. Lactoferrin was released at 25% in the first hour and 84% in 24 h. The nanosuspension formulation speeds drug dissolution and diffusion into the release medium, as shown by the initial rapid release and steady increase in drug release. BLF-NS is a promising candidate for sustained ocular drug delivery because the nanosuspension maintains a stable drug concentration.

##### Ex Vivo Permeation Study

Figure 5b shows the Lactoferrin (BLF) penetration across the cornea was assessed in the ex vivo permeation study for several formulations, including pure BLF, and BLF-NS. Just 35% of lactoferrin was released after 24 h; thus, the BLF formulation displayed a slow and limited release. This implies limited penetration across the cornea, most likely resulting from the absence of a controlled release system or excipients improving drug diffusion. Pure BLF’s slow-release profile suggests that the medication cannot sustainably be effective since it cannot maintain high enough concentrations at the corneal surface. BLF’s slow release can be explained by its lack of a formulation matrix meant to enable increased permeability. Pure BLF fails to cross the corneal barrier effectively without the help of a nanosuspension system. With 84% lactoferrin released after 24 h, the BLF-NS formulation displayed a better drug release profile. The solubility and stability of BLF are improved by the NS formulation, which improves penetration across the cornea. Because small particles diffuse more readily through the corneal epithelium, the nanosuspension’s small particle size is a major determinant of corneal permeability.

The small particle size of the NS lets the drug molecules pass more readily across the corneal barrier, thereby increasing the penetration of BLF-NS. Furthermore, the continuous release profile of the nanosuspension guarantees that the medication stays at the ocular surface for a longer length of time, thereby preserving a therapeutic concentration at the site of action. BLF-NS enhances penetration in several ways, making it effective for ocular drug delivery. Small particles improve diffusion across the corneal epithelium, while improved solubility and sustained release keep lactoferrin bioavailable for prolonged therapeutic action. The formulation’s ability to interact with the corneal barrier, possibly through electrostatic interactions or tight junction disruption, may increase ocular sustained drug release and bioavailability.

### 3.3. Stability Study

The stability study of BLF-GLY demonstrated remarkable physicochemical stability over 3 months at both refrigerated (4 °C) and room temperature (25 °C). There were few changes in particle size, polydispersity index, and zeta potential, indicating that the vesicular structure and dispersion quality remained stable over time [80,81]. The formulation exhibited consistently high Entrapment Efficiency, demonstrating a minor decrease from 99% to 97%, which confirms its robust drug-retaining capacity and resistance to degradation or leakage. No significant aggregation or sedimentation of the samples was observed, and the particle measurement results demonstrated stability for three months, both at room temperature and at four degrees Celsius, as shown in Table 5. The findings demonstrate that BLF-GLY functions as a stable and reliable nanocarrier system, effectively encapsulating and delivering lactoferrin, with the potential for long-term pharmaceutical applications [43].

### 3.4. Fabrication of PVP/HPMC Microneedles Loading Freeze-Dried BLF-NS

Biocompatible polymer microneedles (MNs) are used to treat dry eye disease locally. In this study, dissolving microneedles were made from lyophilized BLF-NS in PVP and HPMC. Our PVP/HPMC ratio formulations were MNs1 (15:10), MNs2 (10:15), and MNs3 (5:25), as shown in Table 2. The chosen ratios were used to investigate the effect of polymer composition on drug release and mechanical properties of microneedles. Fast drug release formulations rely on the hydrophilic polymer PVP, which dissolves quickly and forms films. Hydroxypropyl methylcellulose (HPMC), a viscous gel-forming polymer, gradually dissolves and loses its structure and has a lubricant effect for dry eyes. The most PVP-rich formulation, MNs1, should dissolve quickly and deliver BLF into the eye; dry eye patients in need of relief may benefit. MNs2 dissolves moderately, balancing mechanical strength and release speed with a balanced PVP-HPMC ratio. This formulation could be the best choice for the fast onset and slightly prolonged therapy. MNs3, of HPMC, may be dissolved slowly and release lactoferrin slowly. High HPMC content may increase mechanical strength, allowing microneedles to penetrate the ocular surface while maintaining drug availability and reducing chronic dry eye treatment frequency. Lyophilized BLF-NS ensures even stabilization and distribution of drugs within the microneedle matrix throughout the fabrication process. Lyophilization maintains the physicochemical properties of nanosuspension, facilitating straightforward rehydration and injection into the eye using microneedles. Lactoferrin alleviates oxidative stress and surface inflammation associated with dry eye disease [82,83]. Novel and flexible is his use of BLF-NS-loaded PVP/HPMC microneedles for drug delivery to the eyes. Changing polymer composition improves patient adherence and helps to control drug release, thus improving the efficacy of treatment. For eye treatments requiring exact and continuous drug distribution to the eye surface, this approach could have benefits.

#### 3.4.1. Microneedle Characterizations

##### Physical Characterization of Microneedles—Mechanical Strength/Breaking Strength

Microneedles (MNs) ensure mechanical integrity, guaranteeing effective tissue penetration, free from distortion or breakage. In this work, three distinct microneedle formulations (M1, M2, and M3) were tested mechanically. Each array underwent compressive forces of 250 g, 500 g, and 1000 g, respectively. The decrease in microneedle height, following the application of each force, was used as an indirect measure of mechanical robustness. All microneedles had comparable original lengths before force application, ranging from 243 to 248 µm, with base diameters around 91–93 µm, and tip diameters between 13 and 16 µm. These dimensions are suitable for ocular applications, with narrow tips that are easy to insert and relatively wide bases that provide mechanical support. M3 formulation had the highest mechanical strength and minimal needle length reductions under all applied forces (3 µm at 250 g, 9 µm at 500 g, 21 µm at 1000 g). M1 showed the greatest deformation, decreasing by 4 µm at 250 g, 32 µm at 500 g, and 43 µm at 1000 g, indicating lower mechanical resistance. M2 showed intermediate behavior, with moderate deformation as force increased [84,85]. M3’s improved performance is due to its higher content of HPMC (25%), a polymer that increases structural stiffness when combined with PVP. In contrast, M1, which has the lowest HPMC content (10%), had the poorest mechanical performance. This lends support to the hypothesis that increasing the HPMC content in the formulation improves mechanical integrity [5]. These findings confirm that polymer composition is critical in determining the mechanical properties of microneedles. M3’s mechanical robustness makes it the best candidate for applications that require insertion into delicate tissues, such as the ocular surface, where microneedles must maintain shape without bending or breaking under application pressure.

##### Drug Content Studies

Drug content study is a critical parameter for determining the uniformity and efficiency of drug loading in microneedle formulations. This study’s microneedles were designed to contain 600 µg of BLF per array. The findings revealed that the actual drug content varied slightly across formulations, indicating differences in formulation behavior and drug–polymer interactions [86]. The drug concentrations were 95% in M1, 93% in M3, and 88% in M2. Encapsulation efficiency, an essential factor for accurate dosage and therapeutic efficacy, was elevated in all three formulations. The physicochemical interactions with BLF and the composition of the polymer significantly influence drug loading. The enhanced solubility and compatibility of PVP and BLF, which improve microneedle entrapment, suggest that M1, containing 15% PVP and 10% HPMC, is the optimal formulation for BLF incorporation [86]. M2, containing 15% more HPMC and 10% less PVP, exhibited the lowest drug content at 88%. This indicates that elevating the concentration of HPMC may marginally diminish drug loading, potentially due to the heightened viscosity of the polymer solution, leading to inadequate drug dispersion or the creation of a denser matrix, which could restrict drug entrapment during the drying and solidification stages. M3, with the highest HPMC content (25%) and the lowest PVP (5%), still had a high drug content of 93%, indicating that a higher polymer-to-drug ratio does not impede drug incorporation if the formulation method is optimized. Furthermore, the high drug content found in all formulations suggests that the microneedle fabrication process is repeatable and efficient, with minimal drug loss during processing (e.g., casting, drying) [87,88]. Clinical translation requires consistency, especially in micro-dosing applications like ocular delivery and localized cancer therapy, where overdosing and underdosing can have serious therapeutic consequences. The drug content data show that all three microneedle formulations deliver drugs well. M1 has slightly better encapsulation, but M3 has high drug loading and improved mechanical strength, making it a promising candidate for in vivo testing.

#### 3.4.2. Characterization of the Optimized MN

##### Scanning Electron Microscopy (SEM)

**Figure 6:**
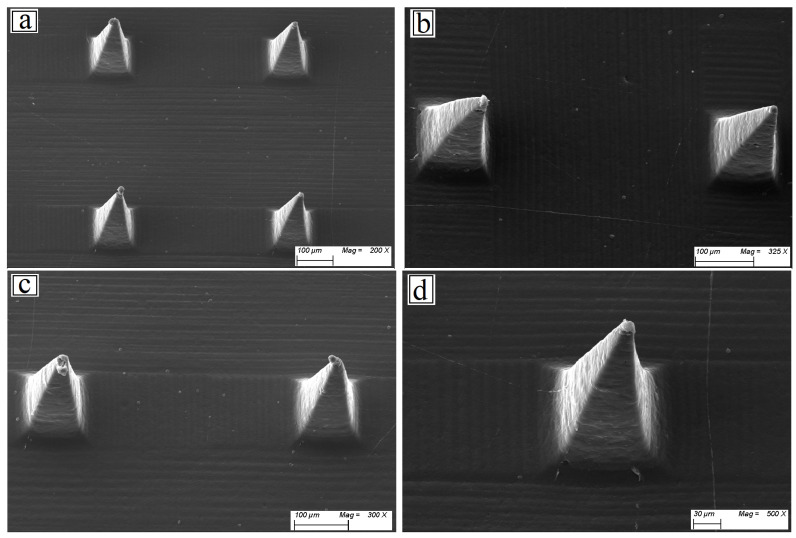
Scanning electron microscopy of optimized PVP/HPMC microneedle loading BLF-NS (MNs3). (**a**) Microneedle array at 200× magnification, scale bar = 100 µm, (**b**) Enlarged view of individual microneedles at 325× magnification, scale bar = 100 µm, (**c**) Closer examination of microneedle tips at 300× magnification, scale bar = 100 µm, and (**d**) High-magnification view of a single microneedle showing detailed surface morphology at 500× magnification, scale bar = 30 µm.

The morphological characteristics of optimized PVP/HPMC microneedles loaded with freeze-dried BLF-NS are shown in Figure 6. MNs integrated with the BLF-NS formulation exhibiting visible pyramidal micro-projections were observed by scanning electron microscopy (SEM) imaging with sharp pyramids [5,89].

##### Fourier Transmission Infrared Spectroscopy (FTIR)

Figure 4a shows Fourier transform infrared spectroscopy for blank MNs and BLF-NS-MNS; the analysis sheds light on the functional groups and molecular interactions found in these formulations, which are vital for the stability and efficacy of the drug delivery system. The blank MN spectra showed different peaks connected to the used excipients in the formulations. Attributed to C-O-C stretching and C-H bending vibrations, the PVP polymer displayed characteristic absorption bands around 1600 cm^−1^ for C=O stretching; HPMC displayed typical peaks in the 1000–1200 cm^−1^ range [90,91]. These peaks also appeared in the FTIR spectra of BLF-NS, implying that PVP and HPMC were part of the microneedle composition. BLF-NS showed lactoferrin-specific peaks, indicating that lactoferrin was successfully incorporated into the microneedle structure. These findings indicate that the microneedles preserved the structural integrity of the lactoferrin protein during formulation, causing minimal disruption to the polymer matrix [92]. The slight shifts and broadening of the amide bands in the BLF-NS and BLF-NS formulations indicate mild interactions between lactoferrin and the polymer matrix, which are likely responsible for the protein’s stability in these formulations [41]. The FTIR analysis demonstrates that lactoferrin maintains its secondary structure and functional groups in both the nanosuspension and microneedle formulations, which is essential for the ocular drug delivery efficacy of the formulations. The results demonstrate that the formulation constituents, including PVP and HPMC, are crucial for stabilizing lactoferrin while enhancing its bioavailability and drug-release characteristics.

##### Differential Scanning Calorimetry (DSC)

Figure 4b shows the Differential Scanning Calorimetry (DSC) for blank MNs, and BLF-NS-MNs; the blank MNs showed a broad endothermic peak at 50–100 °C. This shows the polymer matrix melting point or glass transition temperature. Lactoferrin is not in the blank microneedle MN formulation, and polymeric excipients change temperature, so the MNs curve has no protein-related peaks. The graph for BLF-NS-MNs showed that the microneedle polymer endothermic peaks were layered over protein denaturation peaks. This showed that BLF was successfully added to the microneedle formulation. BLF denaturation peak shifted and broadened again, as seen in the nanosuspension, but this time it was combined with thermal transitions from the MNs matrix [45,55]. This shows that the BLF was successfully incorporated into microneedles, with no significant degradation or loss of structural integrity. The mild shifts and broadening of the protein-related peaks indicate minor interactions between lactoferrin and the excipients; however, these have no significant effect on the protein’s function or stability, despite minor conformational changes caused by interactions with the excipient polymers. These changes are common when proteins are added to drug delivery systems, implying that the lactoferrin is stable and functional in both formulations. An efficient method of delivering drugs to the eyes, the BLF-NS-MNs, manages to include BLF while maintaining thermodynamic stability. The microneedle matrix and the small particle size of the nanosuspension both work together to increase the bioavailability and duration of drug release of BLF, which is crucial for its therapeutic effectiveness.

##### In Vitro Drug Release Q24 of Lactoferrin from BLF-NS-MNs

Figure 5a shows the drug release profiles show that formulation BLF-NS-MNS released 30% of the drug in the first hour and 95% at 24 h, the highest of all tested formulations. The microneedle matrix releases BLF quickly, while the nanosuspension component releases it later. The microneedle system facilitates sustained drug release for 24 h, proving beneficial for therapeutic applications such as dry eye disease. BLF-NS-MNs exhibit an optimal drug release profile characterized by rapid and sustained release. This indicates that microneedles facilitate immediate drug release, whereas nanosuspension extends release duration, rendering it suitable for controlled drug delivery systems. Crucially for the efficacy of ocular drug delivery in both the nanosuspension and microneedle formulations, the FTIR study shows that lactoferrin preserves its secondary structure and functional groups in both forms. The results show that the stabilization of lactoferrin and enhancement of its bioavailability and drug release characteristics depend critically on the components of the formulation, especially PVP and HPMC.

##### Ex Vivo Permeation Study

Figure 5b shows the ex vivo permeation study for BLF across the cornea, several mechanisms can be ascribed to the observed increase in corneal penetration with the microneedle-based formulations; these mechanisms cooperate to improve lactoferrin’s ocular bioavailability and drug delivery. The most efficient system for ocular drug delivery is the BLF-NS-MN formulation since it displayed the highest drug release (93%) at 24 h. Combining microneedles helps this formulation to be beneficial, guaranteeing continuous release of lactoferrin over time. The microneedles allow the medication to be directly penetrated through the corneal epithelium. The temporary microchannels created by the microneedles in the corneal epithelium let the drug pass across the barrier more readily. These tiny holes disturb the tight junctions between epithelial cells, so they enhance the drug’s capacity to diffuse into other layers, including the corneal stroma. Particularly as lactoferrin is an ionic protein, microneedles may also improve penetration through electrostatic interactions with the lactoferrin molecules. Through their interaction, the electrically charged microneedles and lactoferrin can help to retain drugs on the microneedle surface, enhancing their adhesion to the cornea and guaranteeing a higher local concentration at the target site. Microneedles combined with nanosuspension in BLF-NS-MNS produce a synergistic effect, where the microneedles guarantee continuous release, facilitating immediate release [93,94]. This twin mechanism guarantees that lactoferrin stays at the ocular surface for a long period and maximizes corneal penetration, thus extending therapeutic effects. In comparison, the BLF-NS formulation achieved moderate permeation (~85%), while the conventional BLF formulation showed the least permeation (~33%) over the same period. The statistically significant differences between the different formulations underscore the enhanced performance of microneedle-based systems. Key roles in raising the efficacy of drug delivery systems are played by the mechanisms of enhancement, including microchannel development, electrostatic interactions, and the synergistic effect of microneedles and nanosuspension. These results imply that the BLF-NS-MNS formulation is the most promising system for ocular drug delivery since it provides fast initial release and continuous therapeutic concentrations, making it suitable for conditions needing continuous ocular treatment.

### 3.5. Antimicrobial Study

Table 6 shows the antimicrobial activity of Bovine Lactoferrin (BLF) and its optimized nanosuspension formulation (BLF-NS) against selected pathogenic strains: Methicillin-Resistant *Staphylococcus aureus* (*MRSA*), *Staphylococcus aureus*, and *Aspergillus niger* to standard antibiotics, Gentamycin for bacteria and Ketoconazole for fungi. Antimicrobial efficacy was measured using both the zone of inhibition and minimum inhibitory concentration (MIC).

**Figure 7:**
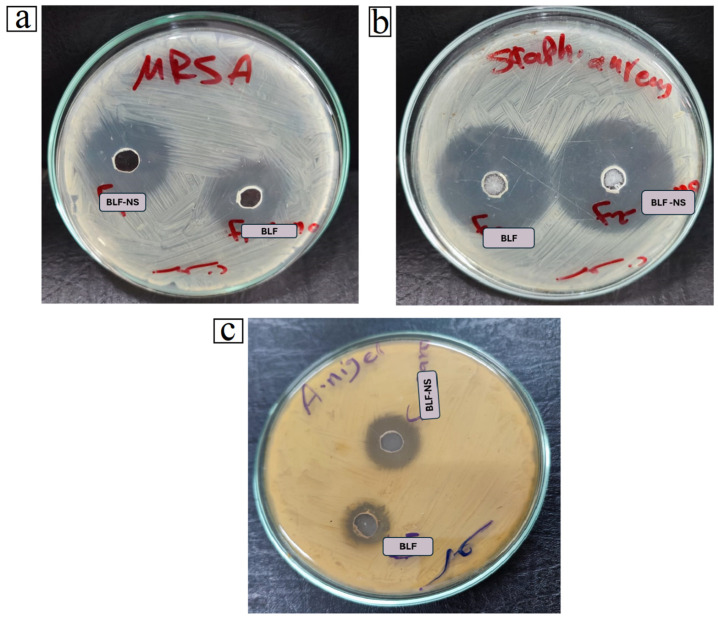
Antibacterial effect of BLF, and BLF-NS against (**a**) Methicillin-Resistant *Staphylococcus aureus*, (**b**) *Staphylococcus aureus*, and (**c**) *Aspergillus niger*. Note: Lactoferrin (BLF), and optimized BLF-loaded nanosuspension (BLF-NS).

The results clearly show that BLF-NS had higher antimicrobial activity than both free BLF and standard antibiotics, particularly against *MRSA* and *Aspergillus niger*. BLF-NS inhibited *MRSA* more effectively (20 ± 0.23 mm) than BLF (18 ± 0.81 mm) and Gentamycin (15 ± 0.37 mm). The MIC value decreased significantly from 400 ± 12.71 µg/mL (control) to 31.25 ± 0.13 µg/mL with BLF and then to 15.63 ± 0.56 µg/mL with BLF-NS. This demonstrates a significant dose-dependent increase in antimicrobial activity via nanoformulation. *S. aureus* exhibited the highest sensitivity to BLF-NS, with the largest inhibition zone (35 ± 0.23 mm). However, the MIC remained consistent across all treatments at 10 µg/mL, indicating that the bacteriostatic effect was already optimal with the control strain, as shown in Figure 7.

For *Aspergillus niger*, an interesting trend emerged, while the standard antifungal ketoconazole and BLF had similar MIC values (500 µg/mL), BLF-NS significantly reduced the MIC to 62.5 µg/mL. The zone inhibition increased from 13 ± 0.94 mm (BLF) to 17 ± 0.13 mm (BLF-NS), suggesting that BLF-NS enhances antifungal efficacy through improved solubility, bioavailability, or cellular penetration.

A variety of factors are likely to contribute to BLF-NS’s improved antimicrobial performance. First, the nanosuspension format increases the surface area-to-volume ratio, allowing for better interaction with microbial cell walls. Previous research, such as that conducted by Ong R et al. (2022), has shown that nanoformulations of lactoferrin penetrate biofilms and cell membranes more effectively, resulting in increased antimicrobial activity [95]. Lactoferrin’s iron-chelating property depletes microbes of essential iron, disrupting vital enzymatic activities and causing cell death. Furthermore, nanoscale delivery systems can improve the stability and controlled release of bioactives such as lactoferrin, as noted by Tong J et al. (2025) [96], thereby increasing antimicrobial efficacy over time. Furthermore, the increased antifungal activity observed against *Aspergillus niger* could be attributed to nanosized lactoferrin’s ability to interfere with spore germination and hyphal growth more effectively than the free form. Previous studies, such as those conducted by Ramamourthy G et al. (2025), have demonstrated lactoferrin’s ability to interact with fungal membranes and induce oxidative stress, mechanisms that may be amplified by nanoencapsulation [97].

Beyond systemic and topical antimicrobial applications, lactoferrin and its nanoformulations show great promise in ocular therapy, particularly for dry eye disease and eye infections. Lactoferrin is a naturally occurring glycoprotein found in human tears that helps to stabilize the tear film, provide anti-inflammatory defense, and protect against microbes. In dry eye, low tear lactoferrin levels are associated with increased inflammation and susceptibility to microbial colonization. BLF, particularly BLF-NS, may play a dual role in treating both the inflammatory and infectious aspects of dry eye. Its iron-chelating activity reduces oxidative stress in ocular tissues, while its antimicrobial action protects the eye from opportunistic pathogens commonly found in DED patients. Several studies, including Carney F et al. (2009), suggest that topical lactoferrin can reduce ocular surface inflammation and improve epithelial healing [98]. The incorporation of BLF into nanosuspensions, such as BLF-NS, is expected to improve corneal penetration, extend ocular surface residence time, and sustain drug release, making it an ideal candidate for advanced ocular drug delivery systems.

In infectious eye diseases, such as bacterial conjunctivitis and keratitis, which are frequently caused by *S. aureus*, *P. aeruginosa*, or fungal pathogens, such as *A. niger*, BLF-NS could be a powerful alternative or adjunct to conventional antimicrobials. BLF-NS’s demonstrated ability to lower MIC values and enlarge inhibition zones supports its potential for treating multidrug-resistant ocular pathogens while reducing the risk of antibiotic resistance development.

Finally, the results support the potential of BLF-NS as a broad-spectrum antimicrobial agent. Its efficacy against both bacterial and fungal pathogens, particularly multidrug-resistant strains like *MRSA* and fungi like *Aspergillus niger*, demonstrates its potential in alternative or adjunctive therapy. The use of BLF-NS in ophthalmology could be a novel therapeutic strategy for treating both dry eye disease and ocular infections by combining anti-inflammatory, antioxidant, and antimicrobial effects in a single nanocarrier platform. These findings support and expand previous research on lactoferrin’s therapeutic potential, emphasizing the role of nanotechnology in maximizing its clinical applications.

### 3.6. In Vivo Study

#### 3.6.1. Eye Irritancy Test (Draize Test)

**Figure 8:**
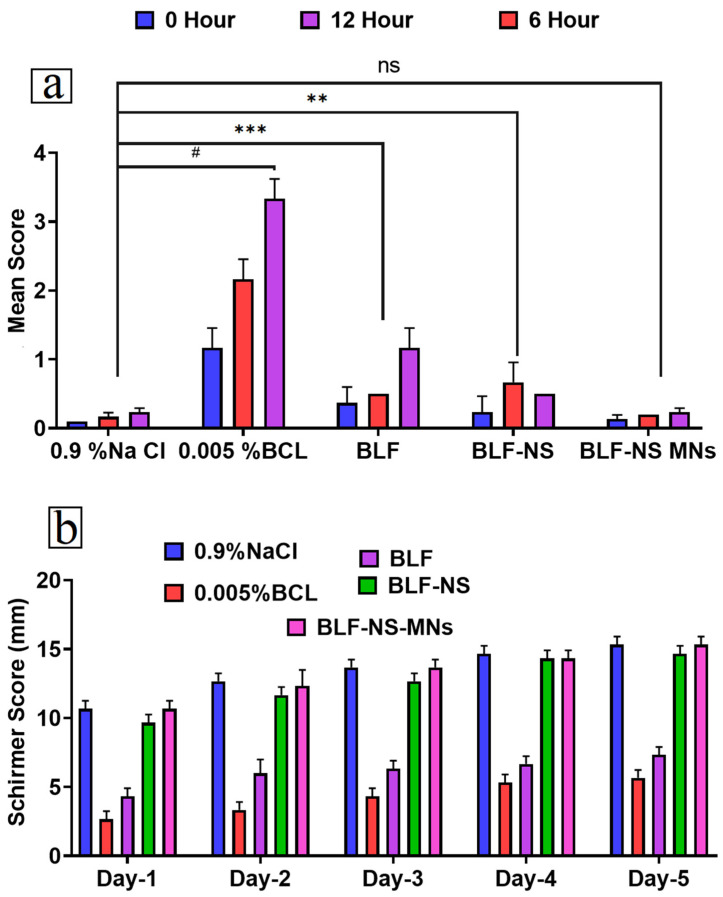
Graphical representation of (**a**) Draize score of 0.9% of NaCl, 0.005% BCL, BLF, BLF-NS, and BLF-NS-MNS, respectively, and (**b**) Schirmer tear score carried out for 5 days. Data are Mean ± SD: ** *p* < 0.01, *** *p* < 0.001, and # *p* < 0.0001 versus the control group; ns (not significant) *p* > 0.05.

The BLF-NS group showed neither redness, excessive tear secretion, nor abnormal blinking characteristics in the treated rabbit eye (Figure 8a). Meanwhile, the control (BLF-solution) group had mild effects on the treated rabbit eye. This indicated that the formulation (BLF-NS) scored 0 according to the Draize scoring system. In addition, there were no significant changes that occurred in either the control 0.9% NaCl-treated or the BLF-NS groups. On the other hand, a significant difference (*p* < 0.0001) in the scores was observed between BLF and BLF-NS-treated groups. This result indicated the high tolerance of BLF-NS in the ocular surface. Using the Draize test in rabbits, the ocular irritancy potential of the optimal BLF-NS formulation was evaluated and then matched to free BLF and 0.9% NaCl (saline) as the negative control. Figure 8a shows each group’s mean irritation scores at 0-, 6-, and 12 h following installation. The irritation scores exhibited a consistently low trend across all groups at each assessed time point, with values varying from roughly 0.03 to 0.23. After 12 h, the BLF group exhibited the highest mean scores at 0.23, while the BLF-NS group followed with a mean score of 0.17. The NaCl group consistently demonstrated the lowest scores, ranging from 0.03 to 0.17. The error bars demonstrate considerable overlap across groups and time points, and the statistical analysis indicated no significant changes (ns) between groups at any individual time point. These findings indicate that the BLF and BLF-NS formulations are well tolerated and do not induce significant ocular irritation in comparison to the saline control. This result is per prior research that has shown lactoferrin is a naturally occurring component of tears and is generally non-irritating to the ocular surface [99]. Additionally, the nanosuspension (BLF-NS) did not exacerbate irritation, suggesting that the nanocarrier system maintains ocular compatibility and safety. The potential safety of BLF-NS as a therapeutic alternative for ocular diseases and dry eye is supported by the low irritation ratings and absence of side effects, which indicate that it is suitable for ophthalmic use. These findings are consistent with prior research that has demonstrated that nano-formulated ocular treatments enhance drug delivery without compromising the safety of the ocular surface.

#### 3.6.2. Schirmer Tear Test (Measurement of Tear Secretion)

Figure 8b presents five days of tear production, quantified by Schirmer scores, across the following treatment groups: 0.9% NaCl, 0.005% BCL, BLF, BLF-NS, and BLF-NS-MNS. Tear production is a critical parameter in the evaluation of dry eye disease (DED), and these results offer valuable insights into the therapeutic efficacy of the formulations. The BLF-NS-MNs group had the highest tear production of the BLF-based treatments, with Schirmer scores gradually increasing from Day 1 to Day 5, eventually reaching levels comparable to the 0.9% NaCl control group. This increased tear secretion demonstrates the therapeutic benefits of the microneedle-based nanosuspension system. On Day 1, BLF-NS-MNS scored significantly higher than BLF and 0.005% BCL, indicating early efficacy. For five days, the BLF-NS-MNs and BLF-NS groups showed consistent improvements in tear secretion, indicating that the nanosuspension and microneedles’ improved permeation and sustained delivery effectively alleviate DED symptoms. Better performance of the BLF-NS-MNs formulation can be ascribed to enhanced corneal penetration, reduced ocular surface inflammation, and higher bioavailability of lactoferrin. Microneedles can also produce reflex tears as a localized ocular reaction to minor mechanical stimulation. Although the BLF group shed somewhat more tears, the BLF-NS and BLF-NS-MN groups greatly exceeded it [100,101]. The results unequivocally demonstrate that in a DED model, BLF-NS-MNs significantly enhance tear production. This outcome endorses the clinical utilization of the formulation as it provides both prolonged therapeutic effects and immediate relief in the management of dry eye syndrome.

#### 3.6.3. mRNA Expression in Cornea and Conjunctiva Detected by qRT-PCR

The therapeutic potential of BLF-based formulations in a dry eye disease (DED) model, as well as the relative expression levels of key genes involved in inflammation, oxidative stress, and lipid metabolism, were assessed: PPARA, COX-2, and SOD 1.

**Figure 9:**
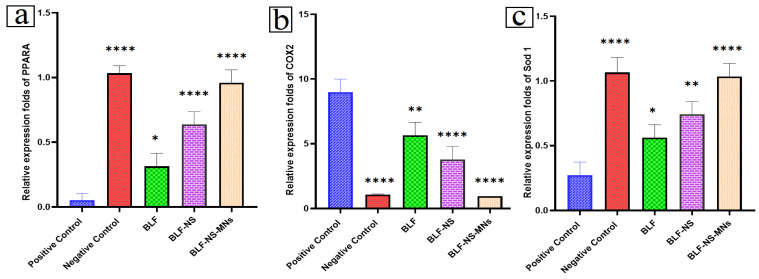
Inhibition of eye inflammation in dry eyes via qRT-PCR analysis demonstrating the relative transcription levels of PPARA (**a**), COX-2 (**b**), and Sod 1 (**c**) (*n* = 6). Data are Mean ± SD: * *p* < 0.05, ** *p* < 0.01, and **** *p* < 0.0001 versus the control group. Note: Group I: normal group (negative control), Group II: diseased group (positive control), Group III: Dry eye treated with BLF solution, Group IV: dry eye treated with BLF-NS, and Group V: dry eye treated with BLF-NS-MNs.

Figure 9a shows that PPARA (Peroxisome proliferator-activated receptor alpha) plays a crucial role in lipid metabolism and maintaining ocular surface homeostasis. In the DED model, the positive control group (DED-induced, untreated) showed significantly lower PPARA expression, indicating disruption of normal metabolic function on the ocular surface. PPARA was expressed at the highest level in the healthy negative control group. BLF-NS-MNs showed the most notable recovery in PPARA expression among the treated groups, almost bringing it back to normal levels; BLF-NS and BLF followed in second importance. This implies that improved lipid control and cellular homeostasis in ocular tissue are achieved by enhanced drug delivery using microneedles coupled with nanosuspension.

Figure 9b shows COX-2, a pro-inflammatory enzyme usually upregulated in DED ocular surface inflammation. Cyclooxygenase-2, consistent with an inflammatory condition, displayed notably high COX-2 expression in the positive control group. Conversely, the negative control group expressed very low COX-2, suggesting a non-inflamed, healthy ocular surface. Even surpassing BLF-NS and BLF, therapy with BLF-NS-MNs produced the most notable decrease in COX-2 expression. The strong anti-inflammatory action of BLF-NS-MNs suggests that lactoferrin, known for its immunomodulating properties, is probably released continuously and with great corneal penetration. The data strongly support that BLF-NS-MNs can effectively reverse inflammatory damage associated with dry eyes. Figure 9c shows that SOD 1 (Superoxide Dismutase 1) is a vital antioxidant enzyme that protects cells against oxidative damage, which is a major contributor to DED pathogenesis. With much reduced SOD 1 expression, the positive control group revealed oxidative stress at the ocular surface. Comparable to the healthy (negative control) group, therapy with BLF-NS-MNs produced a total restoration of SOD 1 expression levels. Though to a lesser degree, BLF-NS and BLF also raised SOD 1 expression. This implies that BLF-NS-MNs efficiently fight oxidative stress, most probably using enhanced delivery and long-term action of lactoferrin, with well-documented antioxidant activity. With notable anti-inflammatory, antioxidant, and homeostasis-restoring effects in the DED model, overall, the gene expression profiles show BLF-NS-MNs are the most effective formulation. Better drug retention, microneedle permeation, and controlled release via nanosuspension most certainly help BLF-NS-MNS to be superior. Targeting the fundamental pathogenic aspects of inflammation and oxidative damage, these results establish BLF-NS-MNs as a potential therapeutic approach for dry eye disease. The dual therapeutic effect of BLF-NS-MNs has been demonstrated by their modulation of anti-inflammatory/antioxidant genes (PPARA, SOD 1) and pro-inflammatory cytokines (TNF-α, IL-6, IL-1β, MCP-1), thereby improving reactive oxygen species (ROS) detoxification and downregulation of NF-κB-mediated inflammatory pathways, which leads to reduced expression of pro-inflammatory cytokines. This combined anti-inflammatory and antioxidant action helps reduce oxidative stress and inflammation related to dry eye conditions.

#### 3.6.4. Inflammatory Cytokines in Conjunctival Tissue Detected by ELISA

**Figure 10:**
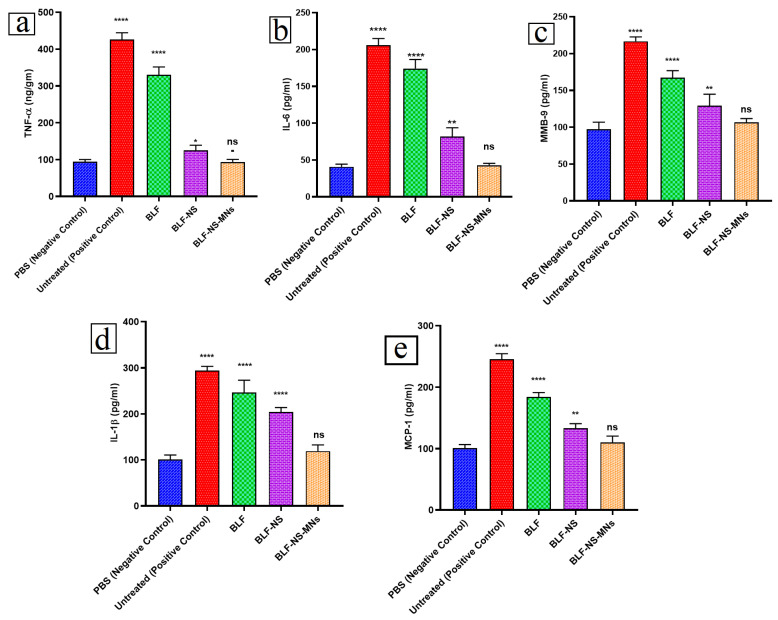
Inhibition of eye inflammation in the dry eye via ELISA analysis demonstrating the relative transcription levels of TNF-α (**a**), IL-6 (**b**), MMB-9 (**c**), IL-1β (**d**) and MCP-1 (**e**) (*n* = 6). Data are Mean ± SD: * *p* < 0.05, ** *p* < 0.01, and **** *p* < 0.0001 versus the control group; ns (not significant) *p* > 0.05. Note: Group I: normal group (negative control), Group II: diseased group (positive control), Group III: dry eye treated with BLF solution, Group IV: dry eye treated with BLF-NS, and Group V: dry eye treated with BLF-NS-MNs.

The pathophysiology of dry eye disease (DED) revolves mostly around inflammation. Using ELISA, the expression levels of important pro-inflammatory cytokines TNF-α, IL-6, MMP-9, IL-1β, and MCP-1 were assessed to evaluate the anti-inflammatory potential of several BLF formulations. As shown in Figure 10a–e, the results provide important new perspectives on how each formulation controls inflammatory responses at the ocular surface.

Figure 10a shows that TNF-α is a major pro-inflammatory cytokine involved in tissue damage and immune activation. The positive control group (DED-induced, untreated) showed a significant increase in TNF-α levels compared to the negative control (PBS-treated), confirming the successful induction of inflammation [102]. TNF-α levels dropped significantly after treatment with BLF-NS-MNs and nearly returned to normal (ns: not significant compared to negative control). BLF-NS also showed a clear decline, though not as much. When BLF was used by itself, it only partially blocked TNF-α. This shows that BLF-NS-MNs have better anti-inflammatory effects, probably because they are more bioavailable and penetrate deeper. Figure 10b shows that IL-6 is another critical mediator in DED, promoting chronic inflammation and epithelial cell dysfunction. The positive control exhibited significantly elevated IL-6. BLF-NS-MNs significantly downregulated IL-6 expression to near-normal levels (ns, not significant), whereas BLF-NS and BLF produced moderate but statistically significant reductions. This trend reinforces the therapeutic potential of microneedle-assisted delivery for suppressing inflammatory signaling. Figure 10c shows that MMP-9 is involved in extracellular matrix degradation and epithelial barrier disruption in DED. The fact that the positive control group had a lot of MMP-9 shows that it plays a part in how the disease worsens. BLF-NS-MNs demonstrated the most effective reduction in MMP-9 levels, bringing them to near-normal ranges. After the BLF-NS and BLF treatments, there was a reduction in MMP-9 levels; however, this decrease was not as pronounced as the one observed following the microneedle-assisted formulation. The decrease in MMP-9 expression indicates a recovery of epithelial integrity and enhancement of barrier function. Figure 10d shows that IL-1β is a potent initiator of inflammation, often elevated in DED. The positive control group had the highest IL-1β levels. BLF-NS-MNs significantly lowered IL-1β levels, again showing anti-inflammatory superiority, with levels statistically indistinguishable from the PBS-treated group. BLF and BLF-NS showed partial reductions. This further supports the role of BLF-NS-MNs in reducing inflammatory signaling at the ocular surface. Figure 10e shows that MCP-1 plays a role in immune cell recruitment and chronic inflammation. Expression peaked in the positive control group. The treatment with BLF-NS-MNs brought MCP-1 levels back to where they were before, followed by BLF-NS and BLF. This means that immune cells are not reaching as deep, and the inflammation is gone. Based on the ELISA results, BLF-NS-MNs is the best formulation for lowering inflammation in DED. All pro-inflammatory cytokines examined were significantly reduced to near-normal levels [43]. The improved corneal delivery and consistent release provided by the microneedle-nanosuspension hybrid system are responsible for this effect. The findings highlight the therapeutic efficacy of BLF-NS-MNs in treating DED by modulating inflammation, repairing epithelial cells, and controlling immune response.

### 3.7. Histopathological Examination

**Figure 11:**
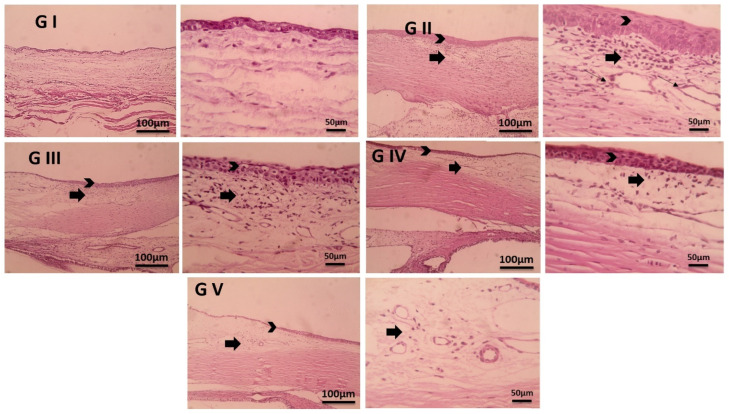
Microscopic pictures of the conjunctiva from a control normal showing normal arrangement and histology. Meanwhile, the conjunctiva from the Diseased Group was subjected to dry eye, showing increased thickness of the epithelial layer (arrowheads), edema, dilated lymphatics (thin black arrows), and many inflammatory cell infiltration in the subconjunctival space (thick black arrows). Conjunctiva from treated Group III showing decreased thickness of the epithelial layer (arrowheads), edema with some inflammatory cell infiltration in the subconjunctival space (thick black arrows). Conjunctiva from treated Group IV showing more decrease in thickness of the epithelial layer (arrowheads), and edema with fewer inflammatory cell infiltration in the subconjunctival space (thick black arrows). Conjunctiva from treated Group V showing normal thickness of the epithelial layer (arrowheads), edema with very few inflammatory cell infiltration in the subconjunctival space (thick black arrows). Low magnifications: ×100, bar 100 μm; and high magnifications: ×400, bar 50 μm.

Figure 11 shows histological sections of conjunctival tissues stained with hematoxylin and eosin, highlighting structural differences between treatment groups in a dry eye disease (DED) model. These images reveal important details about inflammation, tissue remodeling, and the healing potential of various BLF-based formulations. The conjunctival tissues in the normal control group (Group I) had a healthy architecture, with a homogeneous epithelial layer, well-organized stromal tissue, and no inflammation. This group represents the normal physiological condition of the ocular surface, free of any pathogenic modifications. In contrast, the positive control group (Group II), which received no treatment following DED induction, displayed substantial pathological changes. These included a significantly thickened epithelial layer (indicated by arrowheads), marked edema, dilated lymphatic vessels (thin arrows), and heavy infiltration of inflammatory cells in the subconjunctival tissue (thick arrows). These characteristics are typical of chronic ocular surface inflammation and confirm that the dry eye model was successfully induced. Group III, which received only BLF, showed modest improvement. The epithelial thickness was slightly reduced compared to the untreated group, but it remained noticeably hypertrophic. Edema and inflammatory infiltration appear to have persisted, indicating that BLF may be able to help reduce inflammation in certain situations, but its effectiveness may be limited by its low bioavailability in the eye [5,59]. Group IV that received BLF nanosuspension (BLF-NS) improved the most. The epithelial thickness and edema were even lower in this group, as shown by the histological sections. The number of infiltrating inflammatory cells was lower than in the BLF group, indicating that the nanosuspension provided better drug penetration and longer-lasting action. The most notable histological restoration was seen in Group V, which received BLF-NS-MNs (microneedle-assisted nanosuspensions). The epithelial layer in this group was very similar to that in the normal control group, with restored normal thickness and structure [5]. Edema was minimal, and inflammatory cell infiltration was almost nonexistent. These findings highlight the superior efficacy of the microneedle-enhanced formulation, which most likely allowed for deeper corneal penetration and sustained drug release, resulting in effective inflammation resolution and tissue repair. In conclusion, the histological findings strongly support the therapeutic potential of BLF-NS-MNs in DED treatment. This formulation outperformed both BLF and BLF-NS in terms of epithelial damage reversal and inflammation reduction, demonstrating the importance of delivery strategy in ocular drug therapy.

**Figure 12:**
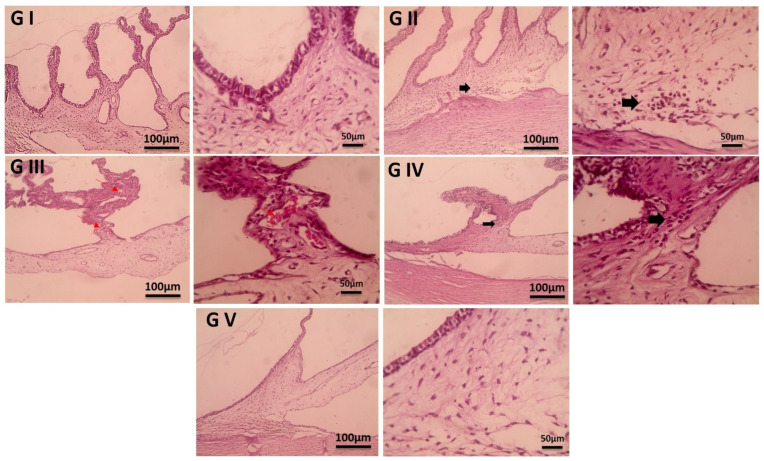
Microscopic pictures of the ciliary body from a control normal showing the normal arrangement and histology of epithelium with no inflammation. Meanwhile, the ciliary body from the Diseased Group was subjected to dry eye, showing some inflammatory cell infiltration (thick arrows). Ciliary body from the treated Group III showing congested blood vessels (red arrow). The ciliary body from the treated Group IV showing very few inflammatory cell infiltration in the underlying layer (thick arrows). The ciliary body from the treated Group V showing a retained normal histological appearance. Low magnifications: ×100, bar 100 μm; and high magnifications: ×400, bar 50 μm.

Figure 12 shows histological sections of the ciliary body, stained with hematoxylin and eosin, derived from multiple experimental groups. In the control group (Group I), the ciliary body demonstrates typical histological structure and intact epithelial morphology; no evidence of inflammatory cell invasion, vascular congestion, or stromal disturbance is present; the epithelial lining remains intact. This is the normal portrayal of ocular tissue in health. By contrast, the positive control group (Group II), which underwent DED induction, displayed clearly pathological changes. Most importantly, there was a clear invasion of inflammatory cells in the underlying connective tissue (highlighted by thick arrows), suggesting an immune response and ciliary body structural damage resulting from persistent ocular surface inflammation. The tissue section from Group III, which only had BLF, showed clogged blood vessels (highlighted by red arrows), which means that the vessels grew and there was a small amount of inflammation. Although some structural integrity was maintained, the existence of vascular congestion shows that BLF by itself offers little defense against DED-induced inflammation in the ciliary area. Treating Group IV with BLF nanosuspension (BLF-NS) produced more encouraging results. This group demonstrated only minor inflammatory cell infiltration in the underlying tissue (thick arrows), with epithelial layers intact. This decreased inflammatory response is due to improved drug permeation and therapeutic activity of the nanosuspension in mitigating inflammatory damage. Group V had the best results when given BLF-NS-MNs. The ciliary body in this group retained its normal histological structure, which was very similar to that of the control group, and successfully repaired tissue integrity while reducing inflammation, as evidenced by the lack of vascular congestion, inflammation, or epithelial disruption. A histological examination of the ciliary body reveals that BLF-NS-MNs offer the best tissue protection and recovery in a DED model. Compared to other groups, this formulation most effectively reduced inflammation while preserving structural integrity, highlighting the benefits of microneedle-assisted delivery in improving therapeutic outcomes for ocular disorders.

## 4. Conclusions

The formulated lactoferrin-loaded nanosuspension (BLF-NS), integrated into dissolving microneedles (BLF-NS-MNs), exhibits considerable promise as a sophisticated therapeutic system for addressing dry eye disease (DED). The research effectively optimized the nanosuspension formulation, attaining a substantial drug loading capacity and improved ocular bioavailability, essential for the management of DED. The microneedle system improved corneal drug penetration, sustained lactoferrin release, and anti-inflammatory and antioxidant effects, as shown by gene expression and cytokine regulation. In vivo Schirmer Tear Test, BLF-NS-MNs produced significantly more tears than other groups, with Schirmer scores approaching normal levels, indicating improved ocular surface hydration and the potential to treat dry eye disease. BLF-NS-MNs released 93% lactoferrin at 24 h, compared to 85% of BLF-NS and 33% from free BLF, in ex vivo permeation studies. Increased penetration and sustained release are due to microneedles creating temporary microchannels in the corneal epithelium to aid drug diffusion and retention. Moreover, mRNA expression studies showed a significant increase in PPARA and SOD 1 together with a downregulation of COX-2, thereby supporting the effective restoration of lipid metabolism, reduced oxidative stress, and lowered inflammation by BLF-NS-MNs. The levels of cytokines (TNF-α, IL-6, MMP-9, IL-1β, and MCP-1) were much reduced, approaching normal values in the BLF-NS-MNs-treated group, so enhancing their anti-inflammatory and antioxidant properties. The BLF-NS-MNs formulation exhibited superior ocular tolerance with negligible irritation, as indicated by Draize test outcomes, positioning it as a viable option for the long-term management of dry eye disease (DED). The integration of nanosuspension and microneedles effectively resolved issues in ocular drug delivery, enhancing sustained therapeutic effects and improving bioavailability, penetration, and drug release, thereby potentially offering significant relief for patients with dry eye disease

## Figures and Tables

**Table 1:** Constructed a 3^4^ Full Factorial Design and its Dependent Responses for the optimization of BLF-NS formulae.

**Factors (Independent Variables)**	**Levels**		
A: Polymer Conc (*w*/*v*%)	1	2	4
B: Stirrer time (h)	0.5	1	2
C: Polymer Type	P188	TPGS	Soy lecithin
D: Plasticizer Type	PG	PEG	Glycerol
**Responses (Dependent Variables)**	**Constraints**		
Y1: P.S (nm)	Minimize		
Y2: P.D.I	Minimize		
Y3: Drug content	Maximize		
Y4: Z.P	Maximize (absolute value)		

Abbreviations: P188, Poloxamer 188; TPGS, D-α-tocopheryl polyethylene glycol succinate; PG, Propylene glycol; PEG, polyethylene glycol; P.S, particle size; P.D.I, polydispersity index; Z.P, zeta potential.

**Table 2:** Fabrication of PVP/HPMC loading lyophilized BLF-NS-MNs.

Formulation	PVP	HPMC
MNs1	15	10
MNs2	10	15
MNs3	5	25

Note: PVP: Polyvinylpyrrolidone, HPMC: Hydroxypropyl Methylcellulose, BLF-NS-MNs: Lactoferrin nanosuspension Microneedles, MNs: Microneedles.

**Table 3:** List of primer sequences used for qRT-PCR.

Gene	Accession Number	Primer Direction	Primer Sequence (5′-3′)	Product Size (bp)	Reference
PPARA	XM_002723354.3	Forward	AGGCCCTCTTCAGAACCTGT	112	[67]
		Reverse	GTGGCTTTCTGTTCCCAGAG		
COX-2	NM_001082388.1	Forward	CGGATTCTACGGTGAAAACTGC	124	[68]
		Reverse	GACGATGTTCCAGACTCCCTTG		
SOD 1	NM_001082627	Forward	ACCTGGGTAATGTGACTGCA	132	[69]
		Reverse	CAATGACACCACAGGCCAAA		
β-actin	NM_001101683	Forward	CGCAGAAACGAGACGAGATT	168	[69]
		Reverse	GCAGAACTTTGGGGACTTTG		

Note: qRT-PCR: quantitative real-time PCR.

**Table 4:** Box–Behnken statistical design for optimization of lactoferrin nanosuspension.

	Factors				Responses			
Run	A: Polymer Concentration (*w*/*v*%)	B: Stirrer Time (h)	C: Polymer Type	D: Plasticizer Type	P.S nm	P.D.I Nm	Z.P mV	Drug Content %
1	1	0.5	P188	PG	480 ± 0.01	0.54 ± 0.03	−32 ± 0.71	75 ± 0.67
2	2	2	P188	PG	650 ± 0.43	0.30 ± 0.09	−28 ± 0.45	70 ± 0.87
3	2	1	P188	PEG	420 ± 0.45	0.25 ± 0.01	−34 ± 0.91	80 ± 0.61
4	4	0.5	P188	PEG	590 ± 0.97	0.20 ± 0.06	−31 ± 0.58	85 ± 0.58
5	2	1	TPGS	PEG	510 ± 0.82	0.22 ± 0.08	−29 ± 0.43	82 ± 0.48
6	4	2	TPGS	PEG	330 ± 0.78	0.65 ± 0.03	−35 ± 0.91	60 ± 0.81
7	1	2	TPGS	PG	310 ± 0.67	0.20 ± 0.01	−30 ± 0.37	68 ± 0.75
8	1	0.5	Soy Lecithin	PEG	540 ± 0.54	0.31 ± 0.03	−27 ± 0.65	55 ± 0.05
9	1	2	Soy Lecithin	PEG	310 ± 0.92	0.23 ± 0.05	−33 ± 0.91	65 ± 0.58
10	4	1	Soy Lecithin	PEG	390 ± 0.89	0.20 ± 0.06	−32 ± 0.56	57 ± 0.91
11	4	2	P188	Glycerol	530 ± 0.82	0.28 ± 0.09	−30 ± 0.46	50 ± 0.78
12	1	0.5	TPGS	Glycerol	420 ± 0.86	0.25 ± 0.02	−31 ± 0.38	85 ± 0.82
13	1	2	P188	Glycerol	500 ± 0.96	0.22 ± 0.03	−32 ± 0.71	80 ± 0.56
14	2	1	P188	PEG	480 ± 0.56	0.20 ± 0.06	−35 ± 0.51	58 ± 0.81
15	2	1	Soy Lecithin	Glycerol	490 ± 0.38	0.20 ± 0.01	−33 ± 0.71	65 ± 0.75
16	4	0.5	Soy Lecithin	Glycerol	410 ± 0.72	0.23 ± 0.01	−32 ± 0.65	83 ± 0.67
17	4	1	TPGS	Glycerol	530 ± 0.67	0.28 ± 0.04	−28 ± 0.91	78 ± 0.84
**18**	**4**	**2**	**P188**	**PEG**	**215 ± 0.45**	**0.21 ± 0.06**	**−28 ± 0.34**	**90 ± 0.66**
19	2	1	Soy Lecithin	PG	605 ± 0.93	0.54 ± 0.02	−27 ± 0.05	77 ± 0.65
20	2	0.5	P188	Glycerol	450 ± 0.71	0.45 ± 0.12	−29 ± 0.43	69 ± 0.35
21	2	2	TPGS	Glycerol	390 ± 0.75	0.65 ± 0.05	−33 ± 0.06	65 ± 0.54
22	4	0.5	TPGS	PG	490 ± 0.47	0.87 ± 0.09	−29 ± 0.34	55 ± 0.72
23	2	0.5	TPGS	PG	450 ± 0.96	0.65 ± 0.05	−30 ± 0.62	61 ± 0.57
24	4	2	Soy Lecithin	PG	554 ± 0.81	0.76 ± 0.03	−32 ± 0.41	59 ± 0.98

Abbreviations: P188: Poloxamer 188, TPGS: D-α-tocopheryl polyethene glycol succinate, PG: Propylene glycol, PEG: polyethylene glycol, P.S: particle size, P.D.I: polydispersity index, and Z.P: zeta potential, and EE%; Entrapment Efficiency. Bolded Run 18: indicated optimized formula. Note: This table summarizes the 3^4^ experimental runs generated by the Box–Behnken Design(BBD) for optimizing BLF-NS. Independent variables were polymer concentration (A: *w*/*v*%), stirring time (B: hours), polymer type (C: P188, TPGS, or soy lecithin), and plasticizer type (D: PEG, PG, or glycerol). Dependent responses included particle size (P.S, nm), polydispersity index (P.D.I), zeta potential (Z.P, mV), and drug content (%). The optimized formulation is highlighted (Run 18). Values are expressed as mean ± SD (*n* = 3).

**Table 5:** The short-term stability results of optimized nanosuspension at 4 °C and 25 °C for 3 months. Mean ± SD (*n* = 3).

Parameters	BLF-GLY Freshly Prepared	BLF-GLY After 3 Months of Storage at 4 °C	BLF-GLY After 3 Months of Storage at 25 °C
P.S (nm)	215 ± 0.45	211 ± 0.05	209 ± 0.81
P.D.I	0.21 ± 0.06	0.21 ± 0.02	0.22 ± 0.01
Z.P (mV)	−28 ± 0.34	−27 ± 0.01	−29 ± 0.46
EE (%)	99 ± 0.66	98 ± 0.98	97 ± 0.46

Note: BLF-GLY: Lactoferrin loaded glycerosomes, P.S: particle size, P.D.I: polydispersity index, Z.P: zeta potential, and EE (%): Entrapment Efficiency.

**Table 6:** The effect of control as ketoconazole for fungi, and Gentamycin for bacteria (control), Lactoferrin (BLF), and optimized BLF-loaded nanosuspension (BLF-NS) on zone inhibition, MIC with the most effective three isolates: Methicillin-Resistant *Staphylococcus aureus*, *Staphylococcus aureus*, and *Aspergillus niger*, respectively.

Strain	Zone Inhibition (mm)			MIC (µg/mL)		
	Control	BLF	BLF-NS	Control	BLF	BLF-NS
(*MRSA*)	15 ± 0.37	18 ± 0.81	20 ± 0.23	400 ± 12.71	31.25 ± 0.13	15.63 ± 0.56
*Staphylococcus aureus*	24 ± 0.23	31 ± 0.43	35 ± 0.23	10 ± 0.12	10 ± 0.04	10 ± 0.62
*Aspergillus niger*	15 ± 0.78	13 ± 0.94	17 ± 0.13	500 ± 0.05	500 ± 0.13	62.50.01

Note. MIC: the minimum inhibitory concentration, ketoconazole for fungi, and Gentamycin for bacteria (control), Lactoferrin (BLF), and optimized lactoferrin nanosuspension (BLF-Ns). Methicillin-Resistant *Staphylococcus aureus* (*MRSA*) ATCC 4330, *Staphylococcus aureus* ATCC 25923, and *Aspergillus niger* (RCMB 002005). Data are reported as the average ± standard deviation obtained from three independent trials (*n* = 3).

## Data Availability

The authors confirm that the data supporting the findings of this study are available within the article.

## Abbreviations

The following abbreviations are used in this manuscript:

BLF	Bovine Lactoferrin
DED	Dry Eye Disease
BCL	Benzalkonium Chloride
BLF-NS	BLF-Nanosuspension
BLF-NS-MNs	BLF Nanosuspension Microneedles
qRT-PCR	Quantitative Real-Time Polymerase Chain Reaction
PPARA	Peroxisome Proliferator-Activated Receptor Alpha
COX-2	Cyclooxygenase-2
SOD 1	Superoxide Dismutase 1
TNF-α	Tumor Necrosis Factor Alpha
IL-6	Interleukin-6
MMP	Matrix Metalloproteinase-9
IL-1β	Interleukin-1 Beta
MCP-1	Monocyte Chemoattractant Protein-1
H&E	Hematoxylin and Eosin
PVP	Polyvinylpyrrolidone
HPMC	Hydroxypropyl Methylcellulose
P.D.I	Polydispersity Index
Z.P	Zeta Potential
MIC	Minimum Inhibitory Concentration
*MRSA*	Methicillin-Resistant *Staphylococcus aureus*
SEM	Scanning Electron Microscopy
FTIR	Fourier Transform Infrared Spectroscopy
DSC	Differential Scanning Calorimetry
HPLC	High-Performance Liquid Chromatography
ANOVA	Analysis of Variance
BBD	Box–Behnken Design
